# Supplementary material for: Hybrid-denovo: a de novo OTU-picking pipeline integrating single-end and paired-end 16S sequence tags
Source: Gigascience. 2017 Dec 15;7(3):gix129. doi: 10.1093/gigascience/gix129 (PMC5841375; doi:10.1093/gigascience/gix129)
Supplement: GIGA-D-17-00280_Original_Submission.pdf [file gix129_giga-d-17-00280_original_submission.pdf]

GigaScience

Hybrid-denovo: A de novo OTU-picking pipeline integrating single-end and paired-end 16S sequence tags

--Manuscript Draft--

Manuscript Number:	GIGA-D-17-00280
Full Title:	Hybrid-denovo: A de novo OTU-picking pipeline integrating single-end and paired-end 16S sequence tags
Article Type:	Technical Note
Funding Information:	Center for Individualized Medicine, Mayo Clinic — Dr. Xianfeng Chen
Abstract:	Background: Illumina paired-end sequencing has been increasingly popular for 16S rRNA gene-based microbiota profiling. It provides higher phylogenetic resolution than single-end reads due to a longer read length. However, the reverse read (R2) often has much significantly base quality and a large proportion of R2s will be discarded after quality control, resulting in a mixture of paired-end and single-end reads. A typical 16S analysis pipeline usually processes either paired-end or single-end reads but not a mixture. Thus, the quantification accuracy and statistical power will be reduced due to the loss of a large amount of reads. As a result, rare taxa may not be detectable with paired-end approach or low taxonomic resolution will be resulted with single-end approach.<br>Findings: To have both the higher phylogenetic resolution provided by paired-end reads and the higher sequence coverage by single-end reads, we propose a novel de novo OTU-picking pipeline, hybrid-denovo, that can process a hybrid of single-end and paired-end reads. Using high quality paired-end reads as a "gold standard", we show that hybrid-denovo achieved the highest correlation with the "gold standard" and performed better than the approaches based on paired-end or single-end reads in terms of quantifying the microbial diversity and taxonomic abundances. By applying our method to a rheumatoid arthritis (RA) data set, we demonstrated that hybrid-denovo captured more microbial diversity and identified more RA-associated taxa than paired-end or single-end approach.<br>Conclusions: Hybrid-denovo is more powerful than de novo OTU picking approaches based on paired-end or single-end 16S sequence tags, and is recommended for 16S rRNA gene targeted paired-end sequencing data.
Corresponding Author:	Xianfeng Chen<br><br>UNITED STATES
Corresponding Author Secondary Information:	
Corresponding Author's Institution:	
Corresponding Author's Secondary Institution:	
First Author:	Xianfeng Chen
First Author Secondary Information:	
Order of Authors:	Xianfeng Chen<br>Stephen Johnson<br>Patricio Jeraldo<br>Junwen Wang<br>Nicholas Chia<br>Jean-Pierre A Kocher<br>Jun Chen

Powered by Editorial Manager® and ProduXion Manager® from Aries Systems Corporation

**Order of Authors Secondary Information:**	
**Opposed Reviewers:**	
**Additional Information:**	
**Question**	**Response**
Are you submitting this manuscript to a special series or article collection?	No
**Experimental design and statistics**<br><br>Full details of the experimental design and statistical methods used should be given in the Methods section, as detailed in our Minimum Standards Reporting Checklist. Information essential to interpreting the data presented should be made available in the figure legends.<br><br>Have you included all the information requested in your manuscript?	Yes
**Resources**<br><br>A description of all resources used, including antibodies, cell lines, animals and software tools, with enough information to allow them to be uniquely identified, should be included in the Methods section. Authors are strongly encouraged to cite Research Resource Identifiers (RRIDs) for antibodies, model organisms and tools, where possible.<br><br>Have you included the information requested as detailed in our Minimum Standards Reporting Checklist?	Yes
**Availability of data and materials**<br><br>All datasets and code on which the conclusions of the paper rely must be either included in your submission or deposited in publicly available repositories (where available and ethically appropriate), referencing such data using a unique identifier in the references and in the "Availability of Data and Materials" section of your manuscript.<br><br>Have you have met the above requirement as detailed in our Minimum Standards Reporting Checklist?	Yes

Manuscript
Click here to download Manuscript bmc_article.tex

RESEARCH

Hybrid-denovo: A de novo OTU-picking pipeline integrating single-end and paired-end 16S sequence tags

Xianfeng Chen[1], Stephen Johnson[1], Patricio Jeraldo[2], Junwen Wang[1], Nicholas Chia[2], Jean-Pierre A Kocher[1] and Jun Chen[1*]

[*]Correspondence: chen.jun2@mayo.edu
[1]Department of Health Sciences Research and Center for Individualized Medicine, Mayo Clinic, 55905 Rochester, USA
Full list of author information is available at the end of the article

Abstract

Background: Illumina paired-end sequencing has been increasingly popular for 16S rRNA gene-based microbiota profiling. It provides higher phylogenetic resolution than single-end reads due to a longer read length. However, the reverse read (R2) often has much significantly base quality and a large proportion of R2s will be discarded after quality control, resulting in a mixture of paired-end and single-end reads. A typical 16S analysis pipeline usually processes either paired-end or single-end reads but not a mixture. Thus, the quantification accuracy and statistical power will be reduced due to the loss of a large amount of reads. As a result, rare taxa may not be detectable with paired-end approach or low taxonomic resolution will be resulted with single-end approach.

Findings: To have both the higher phylogenetic resolution provided by paired-end reads and the higher sequence coverage by single-end reads, we propose a novel *de novo* OTU-picking pipeline, *hybrid-denovo*, that can process a hybrid of single-end and paired-end reads. Using high quality paired-end reads as a “gold standard”, we show that *hybrid-denovo* achieved the highest correlation with the “gold standard” and performed better than the approaches based on paired-end or single-end reads in terms of quantifying the microbial diversity and taxonomic abundances. By applying our method to a rheumatoid arthritis (RA) data set, we demonstrated that *hybrid-denovo* captured more microbial diversity and identified more RA-associated taxa than paired-end or single-end approach.

Conclusions: *Hybrid-denovo* is more powerful than *de novo* OTU picking approaches based on paired-end or single-end 16S sequence tags, and is recommended for 16S rRNA gene targeted paired-end sequencing data.

Keywords: microbiome; OTU picking; 16S rRNA

Findings

Background

The microbiome plays an important role in global ecology, nutrient cycling, and disease [1]. Targeted sequencing of the hypervariable region of the 16S rRNA gene is now routinely used to profile microbiota. Identifying related groups of organisms known as operational taxonomic units or OTUs remains a central part of the analysis of microbiome data. Both *de novo* and reference-based approaches have been proposed for processing 16S rDNA reads - each with complementary strengths and weaknesses. *De novo* OTU-picking naively clusters reads based on sequence similarity. It has the advantages of not requiring any prior knowledge or reference

about the target molecule, and produces OTU groupings that are more naturally aligned to the data. However, *de novo* approaches require comparison of the same gene region. Reference-based approaches can get around this limitation, but rely on a pre-existing set of OTU representatives that may or may not be appropriate for a particular dataset [2].

More specifically, the challenge presented by Illumina paired-end reads is that the reverse read (R2) often has a much lower base quality than the forward read (R1). For the 16S datasets generated at the Mayo Clinic Core Facility, only 24% of R2s passed quality control (QC) between 2013-2015 as opposed to 83% for R1s (Supplementary Fig. 1). We are then left with a smaller set of high-fidelity paired-end reads (R1- R2) and a deeper set of single-end reads (R1). To use a *de novo* OTU picking approach, one would have to choose between the more accurate taxonomic identification using R1-R2 or improved detection of rare taxa using R1[3]. To integrate information from both paired-end and single-end reads, we propose *hybrid-denovo*, a pipeline that combines paired-end and single-end reads in order to retain the advantages of *de novo* OTU-picking while maximizing ability to detect rare taxa.

Methods

Hybrid-denovo first constructs an OTU backbone using only paired-end reads. The single-end reads are mapped to the OTU backbone, creating new OTUs if previously unmapped (Fig. 1A). The same quality control and OTU-picking process as implemented in IM-TORNADO is used to build the OTU backbone [3]. Specifically, quality filtering was performed using Trimmomatic [4] with a hard cutoff of PHRED score Q3 for 5' and 3' ends of the reads, trimming of the 3' end with a moving average score of Q15, with a window size of 4 bases, and removing any remaining reads shorter than 75% of the original read length. Reads with any ambiguous base calls were discarded. Surviving read pairs were further trimmed down to specified cutoffs to uniformize the length of both reads, then concatenated and sorted by cluster size. Afterwards a *de novo* OTU-picking was conducted via UPARSE algorithm [5, 6]. Though UPARSE algorithm has performed *de novo* chimera removal, we additionally used UCHIME [7] to perform a reference based chimera removal against GOLD database (https://drive5.com/uchime/gold.fa), resulting in a set of high quality OTU representatives. We then mapped the single-end R1s to the R1-end of the OTU representatives using USEARCH (if there are multiple hits with the same score, the most abundant one will be chosen by default). The remaining unmapped R1s were clustered into new OTUs via UPARSE algorithm and added to the list of OTUs generated by the paired-end reads. Thus, the OTU representatives consist of a mixture of single-end and paired-end reads. We then aligned all the OTU representatives using the structure alignment algorithm Infernal trained on the Ribosomal Database Project's (RDP) database [8, 9]. OTU representatives that were not aligned or had negative alignment scores were removed since they hypothetically represented non-bacteria. A phylogenetic tree was built from the aligned OTU representatives using FastTree [10]. FastTree has little penalty on end-gaps, which is favorable when processing a mixture of single-end and paired-end reads. Finally, R1 and R2 reads were stitched together with ambiguous nucleotides (a

string of "N"s) in between and then assigned a taxonomy by the RDP classifier [11] trained on the Greengenes database (http://greengenes.secondgenome.com/). OTUs not classified as Bacteria and singleton OTUs were removed as they were presumed contaminants. Note that this step may have lost diversity that is not represented in the database and is a tradeoff between accuracy and completeness. The complete workflow of our pipeline is given in Supplementary Fig. 2.

To validate our approach, we created a "gold standard" data set with high-quality paired-end reads based on the 837 high-coverage human fecal samples sequenced at the Mayo Core Facility (V3-V5 16S amplicon, 694 nt, 357F/926R primers) [12]. These fecal samples were collected from 20 subjects using 6 different methods (no additive, RNAlater, 70% ethanol, EDTA, dry swab, and fecal occult blood test (FOBT)). The samples were immediately frozen or stored in room temperature for four days to study the stability of the microbiota. Each condition had 2-3 technical replicates to assess the reproducibility. We ran Trimmomatic [4] for quality control and trimmed R1s down to 250bp and R2s down to 200bp to ensure high base quality, resulting in non-overlapping paired-end reads. For each sample, we retrieved 8,000 high-quality paired-end reads. We then performed OTU-picking and taxonomy assignment based on these paired-end reads using IM-TORNADO. These resulting OTUs and their associated taxonomy constitute the "gold standard" dataset. We then created three artificial data sets from the "gold standard" with 25%, 50% and 75% of R2 reads remaining. The three data sets represented different levels of R2 quality encountered in practice. We compared *hybrid-denovo* to approaches based on single-end R1s or paired-end reads using the artificial data sets. Performance was evaluated by calculating the Spearman's correlation with the "gold standard" in terms of microbial β-diversity (unweighted and weighted UniFrac, and Bray-Curtis distance) and genus-level relative abundances.

We also compared our pipeline to QIIME and mothur (version 1.8.0 and 1.39.3 respectively) [13, 14] on the "gold standard" data set. Since QIIME and mothur currently do not support *de novo* OTU-picking based on non-overlapping reads, we ran QIIME and mothur on the R1 reads. Parameter settings were chosen to be as comparable to that of *hybrid-denovo*. Since we created good quality-reads by using Trimmomatic, we reduced potential variation in performance between pipelines by not applying additional read QC filters. An RDP classifier trained on Greengenes v13.5 was used to classify reads for all pipelines. Singletons and non-bacteria OTUs (based on taxonomy) were filtered out. The major differences between the three pipelines in addition to the commands used to reproduce the results are documented in Supplementary Note 1. We assessed performance by investigating (1) the number of detected genera and percentage of unclassified reads at the genus level, (2) Mantel correlation using Bray-Curtis matrices, and (3) the intra-class correlation coefficient (ICC) for these core OTUs and genera observed in more than 90% of the samples. ICC is a measure of the correlation between the technical replicates. A high value indicates less measurement error. ICC was calculated using the R ICC package [15].

Finally, we demonstrated the performance of the proposed method on a dataset from the study of the stool microbiome of RA (rheumatoid arthritis) patients, which consists of 40 RA patients and 49 controls (V3-V5 16S amplicon, 694 nt) [16]. We applied DESeq2 to the taxa count data for differential abundance analysis [17] and compared the RA-associated OTUs/genera recovered by different approaches.

Results

The correlation of microbial β-diversity with the "gold standard" was generally high for all the three approaches (Fig. 1B). However, the approach based on single-end R1 tends to have a lower correlation when BC distance was used (the single-end R1 approach was invariant to the R2 quality). The paired-end approach, on the other hand, had a much lower correlation for unweighted UniFrac when only 25% R2s remain. This is due to the fact that unweighted UniFrac captures community membership, which is contributed mainly by rare taxa, and many rare taxa are no longer detectable by the paired-end approach due to loss of reads. In contrast, Hybrid-denovo was very robust and had the best or close to the best correlation with the "gold standard" in both diversity measures. For weighted UniFrac distance, the correlation was similarly high for all the three methods since the weighted UniFrac is most influenced by dominant taxa and all the methods quantify these dominant taxa very well (Fig. 1B).

We next studied the performance of taxonomic profiling of the proposed approach. Based on the 56 genera with prevalence greater than 10%, *hybrid-denovo* had much higher correlation with the "gold standard" across all scenarios considered and its performance was not very sensitive to the percentage of R2 remaining (Fig. 1C). In contrast, the performance of paired-end approach depends strongly on the R2 quality and had much lower correlation when R2 quality was low. The single-end R1 approach was invariant to the R2 quality as expected and performed better than the paired-end approach only when R2 quality was low. Supplementary Fig. 3 showed the individual genus correlations. For the single-end approach, two genera showed zero correlation with the "gold standard" because all of their R1 reads were re-classified at the family level due to their short length (*Lachnobacterium* mapped to *Ruminococcaceae* and *Erwinia* mapped to *Enterobacteriaceae*), indicating the increased phylogenetic resolution using paired-end reads. For the paired-end approach, genera with low-abundance exhibited a lower correlation, indicating the decreased quantification accuracy due to loss of paired-end reads.

We also compared *hybrid-denovo* to mothur and QIIME, the two pre-dominant pipelines for 16S data, based on the "gold standard" data set. Mothur and QIIME took around 24 and 6 hours respectively to complete the analysis of the "gold standard" dataset (n=837), compared to around 1 hour for our pipeline. Mothur and QIIME produced a total of 4,599 and 2,898 non-singleton OTUs respectively while *hybrid-denovo* produced 1,094, 1,086, 1,079 and 1,049 non-singleton OTUs on data sets with different percentages of good quality R2 reads (100%, 75%, 50% and 25%). Though our pipeline resulted in a smaller number of OTUs, we detected a larger number of genera than mothur and QIIME. For example, application of *hybrid-denovo* to the data set with 50% good quality R2 reads yielded a total of 110 genera, compared to 70 and 84 for QIIME and mothur respectively (Fig. 2, upper right, Venn diagram). Using BLAST on the paired-end counterparts of the QIIME and mothur-specific genera (classified based on R1 reads) against the Greengenes database re-assigns many of the reads to other genera. This indicates that those genera were probably misclassified due to shorter reads. Though the genus-level microbiota profiles for the 20 subjects were similar for all the pipelines (Fig. 2), *hybrid-denovo* had a much lower proportion of reads with unknown genus identity

(5%) than mothur and QIIME (14% and 18% respectively). Taken together, these observations demonstrated that *hybrid-denovo* had increased taxonomic resolution due to the use of longer reads. Interestingly, all the pipelines could yield similar inter-sample relationship as measured by Mantel correlation coefficients based on Bray-Curtis distance matrices (Table 1). The availability of technical replicates of the data set allows us to compare different pipelines using intra-class correlation coefficients (ICCs). A high ICC indicates less variability introduced by the bioinformatics pipeline. We calculated the ICCs for different fecal collection methods for the core OTUs and genera, which occurred in more than 90 % of the samples. Our pipeline generally had higher ICCs (less variation between technical replicates) than mothur and QIIME (Fig. 3). In contrast, mothur and QIIME did not perform as well on the core OTUs and genera respectively.

We also applied our method to a dataset from a RA study [16], where about 40% R2s were discarded after quality control (Supplementary Table 1). *Hybrid-denovo* resulted in the largest number of OTUs and genera as expected (Fig. 4A), and covered all genera from paired-end approach and the majority genera from single-end R1 approach (Fig. 4C) There were a total of five R1-specific genera, for example, *Bacteria Firmicutes Clostridia Clostridiales Clostridiaceae 02d0* and *Bacteria Firmicutes Clostridia Clostridiales Clostridiaceae Sarcina* were re-classified to *Bacteria Firmicutes Clostridia Clostridiales Clostridiaceae Clostridium* when their paired-end counterparts were used, indicating that the R1-specific genera were misclassified due to their short read length.

Besides the comparison of the detected genera, we also demonstrated the advantage of *hybrid-denovo* in the context of differential abundance analysis using DESeq2 [17]. We excluded OTUs that occurred in less than 10% samples from testing. A total of 758, 578 and 393 OTUs were tested using *hybrid-denovo*, paired and R1 approaches, respectively. Due to higher read counts and increased phylogenetic resolution, *hybrid-denovo* recovered more differential OTUs (Fig. 4B). We identified a total of 126 significant OTUs at an FDR-adjusted P value of 0.01 compared to 93 and 80 OTUs for paired-end and single-end R1 approaches, respectively. Since different methods had their own definition of OTUs and direct comparison of the differential OTUs is difficult, we instead compared the genus identity of the identified OTUs. The differential OTUs identified by *hybrid-denovo* were classified into 33 genera, in comparison to 32 and 34 for the paired-end and single-end R1 approaches (Fig. 4B). There were 20 significant genera shared by all three methods (Fig. 4D), many of which were reported by previous studies [16, 18, 19]. For example, *Bacteroides* is enriched in control samples, while *Collinsella, Eggerthella, Prevotella* and *Clostridium* are enriched in RA samples. Even though the total number of differential genera were similar for all the methods, *hybrid-denovo* identified the most genera (n=11) that were shared by either one of the other two approaches, compared to 6 and 9 for paired-end and single-end R1 approach, indicating that the *hybrid-denovo* approach was able to identify differential genera that were otherwise missed by either paired-end or single-end R1 approach. Furthermore, *hybrid-denovo* had the least number of method-specific genera (n=2) in contract to paired-end (n=6) and R1 single-end (n=5). The method-specific genera might be less reliable due to lack of the support from other methods. For example, R1 approach found *Veillonella* to be enriched in control samples, which is conflict with a previous study

[18]. Interestingly, among the two of the *hybrid-denovo* specific genera, *Klebsiella*, which was enriched in healthy people, was reported by Zhang et al. [19].

Discussion

We proposed *hybrid-denovo* for *de novo* OTU-picking based on paired-end 16S sequence tags. Through simulations and real data examples, we showed that our approach had better performance than single-end or paired-end approach in quantifying the microbial diversity and taxonomic abundance, due to the full use of the information in the paired-end reads.

Based on the size of 16S amplicons and the length of the paired-end reads, we could have overlapping or non-overlapping paired-end reads. For example, sequencing of the V4 region (252 nt, 515F/806R primers) produces overlapping paired-end reads while sequencing of the V3-V5 region (694 nt, F357/R926 primers) results in non-overlapping paired-end reads using Illumina MiSeq (250 bp $\times$2). Since QIIME and mothur currently do not support *de novo* OTU-picking based on non-overlapping paired-end reads, the main advantage of our pipeline lies in the ability to process non-overlapping paired-end reads. However, our pipeline could also be applied to overlapping paired-end reads by using PANDAseq [20] to stitch the paired-end reads together. It is noted that some existing pipelines could also process a mixture of paired-end and single-end reads with different capacities. For example, the recently proposed LotuS pipeline uses good-quality R1 reads to build OTUs, followed by a post-clustering merging of R1 and R2 to increase the accuracy of the taxonomy [21]. However, the OTU-level resolution is still determined by R1 reads.

There are new pipelines that have been developed for 16S data. It is interesting to benchmark *hybrid-denovo* against these state-of-the-art pipelines. We selected DADA2 and LotuS [21, 22] for comparison since they have been demonstrated to have an overall better performance than QIIME and mothur and have been increasingly used by the community. We repeated the same analysis on the "gold standard" data set with complete read pairs. The specific command lines used for DADA2 and LotuS are documented in Supplementary Note 1. DADA2 produced 18,389 sequence variants (SVs) while LotuS produced 472 OTUs. The Mantel correlation on the OTU/SV-level Bray-Curtis distance is high between *hybrid-denovo* and LotuS (ρ=0.93) but moderate between *hybrid-denovo* and DADA2 (ρ=0.71). Interestingly, the Mantel correlation on the genus-level Bray-Curtis distance is high between all methods (ρ>0.97), indicating all methods could produce similar genus-level profiles (Supplementary Fig. 4). Similar ICC analysis demonstrated that all the methods had relatively high ICCs but *hybrid-denovo* had overall the best performance (Supplementary Fig. 5).

One problem for *de novo* OTU-picking is the potential inflated OTU number, which could be due to sources such as sequencing errors, chimera and environmental contaminant [6]. In *hybrid-denovo*, we used various quality filtering criteria to reduce the number of spurious OTUs. For example, we applied Trimmomatic [4] to trim and remove reads with low base quality, removed reads with any ambiguous bases, removed singleton OTUs, used the Infernal package [8] to remove non-structurally aligned OTUs and used reference-based UCHIME as an additional chimera removal process[6]. However, even these filters might fall short of reducing inflated diversity

estimate due to unknown sequencing errors. Improving the diversity estimate from *hybrid-denovo* will be the focus of our future work.

Competing interests
The authors declare that they have no competing interests.

Acknowledgements
This study was supported by Mayo Clinic Center for Individualized Medicine.

Author details
[1]Department of Health Sciences Research and Center for Individualized Medicine, Mayo Clinic, 55905 Rochester, USA. [2]Department of Surgery, Mayo Clinic, 55905 Rochester, USA.

References

1. Cho I and Blaser MJ, **The human microbiome: at the interface of health and disease**. *Nat. rev. genet.* 2012;13(4):260-70.
2. McDonald D, Birmingham A, and Knight R. **Context and the human microbiome**. *Microbiome* 2015; 3:52.
3. Jeraldo P, Kalari K, Chen X, Bhavsar J, Mangalam A, White B, et al. **IM-TORNADO: A tool for comparison of 16s reads from paired-end libraries**. *PloS ONE* 2014; 9(12):e114804.
4. Bolger MA, Lohse M, and Usadel B. **Trimmomatic: a flexible trimmer for illumina sequence data**. *Bioinformatics* 2014; 30(15):2114-20.
5. Edgar RC. **Search and clustering orders of magnitude faster than blast**. *Bioinformatics* 2010; 26(19):2460-1.
6. Edgar RC. **Uparse: highly accurate OTU sequences from microbial amplicon reads**. *Nat. methods* 2013;10(10):996-8.
7. Edgar RC, Haas BJ, Clemente JC, Quince C, Knight R. **UCHIME improves sensitivity and speed of chimera detection**. *Bioinformatics* 2011;10(10):27(16):2194-200.
8. Nawrocki EP, Kolbe DL, and Eddy SR. Infernal 1.0: inference of RNA alignments. *Bioinformatics* 2009; 25(10):1335-7.
9. Cole JR, Wang Q, Cardenas E, Fish J, Chai B, Farris RJ, et al. **The ribosomal database project: improved alignments and new tools for rRNA analysis**. *Nucleic Acids Res.* 2009;37:D141?D145.
10. Price MN, Dehal PS, and Arkin AP. **Fasttree 2–approximately maximum-likelihood trees for large alignments**. *PloS ONE* 2010; 5(3):e9490.
11. Wang Q, Garrity GM, Tiedje JM and Cole JR. **Naive Bayesian classifier for rapid assignment of rRNA sequences into the new bacterial taxonomy**. *Appl. environ. microbiol.* 2007; 73(16):5261-5267
12. Sinha R, Chen J, Amir A, Vogtmann E, Shi J, Inman KS, et al. **Collecting fecal samples for microbiome analyses in epidemiology studies**. *Cancer epidemiol. biomarkers prev.* 2016; 25(2):407-16.
13. Caporaso JG, Kuczynski J, Stombaugh J, Bittinger K, Bushman FD, Costello EK, et al. **QIIME allows analysis of high-throughput community sequencing data**. *Nat. methods* 2010;7:335-336
14. Schloss PD, Westcott SL, Ryabin T, Hall JR, Hartmann M, Hollister EB, et al. **Introducing mothur: open-source, platform-independent, community-supported software for describing and comparing microbial communities**. *Appl. environ. microbiol.* 2009; 75(23):7537-41.
15. Wolak ME, Fairbairn DJ, Paulsen YR **Guidelines for Estimating Repeatability** *Methods in Ecology and Evolution* 2012; 3(1):129-137
16. Chen J, Wright K, Davis JM, Jeraldo P, Marietta EV, Murray J, et al. **An expansion of rare lineage intestinal microbes characterizes rheumatoid arthritis**. *Genome med.* 2016; 8(1):43
17. Love MI, Huber W, Anders S. **Moderated estimation of fold change and dispersion for RNA-seq data with DESeq2**. *Genome biol.* 2014; 15(12):550
18. Scher JU, Sczesnak A, Longman RS, Segata N, Ubeda C, Bielski C, et al. **Expansion of intestinal Prevotella copri correlates with enhanced susceptibility to arthritis**. *Elife* 2013; 5(2):e01202
19. Zhang X, Zhang D, Jia H, Feng Q, Wang D, Liang D, et al. **The oral and gut microbiomes are perturbed in rheumatoid arthritis and partly normalized after treatment**. *Nat. med.*, 2015, Aug;21(8):895-905
20. Masella AP, Bartram AK, Truszkowski JM, Brown DG, and Neufeld JD. **Pandaseq: paired-end assembler for illumina sequences**. *BMC bioinformatics* 2012; 13:31
21. Hildebrand F, Tadeo R, Voigt AY, Bork P, Raes J. **LotuS: an efficient and user-friendly OTU processing pipeline**. *Microbiome* 2014; 2:30
22. Callahan BJ, McMurdie PJ, Rosen MJ, Han AW, Johnson AJ, Holmes SP. **DADA2: high-resolution sample inference from Illumina amplicon data**. *Nat. methods* 2016;13:581-3

Figures

Figure 1 Overview and evaluation of the Hybrid-denovo approach. A. *Hybrid-denovo* illustration. B. Mantel correlation of β-diversity distance matrices (Unweighted UniFrac, Weighted UniFrac and Bray-Curtis distance) with the "gold standard" for the three approaches at different percentages of good-quality R2 reads. Error bars represent standard errors of the estimate based on 100 bootstrap samples. C. Boxplot of correlations of the relative abundances of 56 prevalent genera with the "gold standard".

Figure 2 Comparison of mothur, QIIME and Hybrid-denovo on genus-level profiles. *Hybrid-denovo* are run on data sets with different percentages of good quality R2 reads (100%, 75%, 50% and 25%). Each column represents the microbiota profile of an individual averaged over all replicates. The overlaps of detected genera between the three pipelines are shown in the Venn diagram.

Figure 3 Comparison of mothur, QIIME and Hybrid-denovo on intra-class correlation coefficients (ICCs) of the core genera (A) and OTUs (B). ICCs are calculated based on the technical replicates for six different fecal collection methods. *Hybrid-denovo* are run on data sets with different percentages of good quality R2 reads (100%, 75%, 50% and 25%).

Figure 4 Comparison of the R1, Paired and Hybrid approaches on the RA dataset. A. Number of detected OTUs (red) and genera (blue). B. Number of significant OTUs (red) and genera (blue) from differential abundance analysis (FDR≤ 0.01). C. Venn diagram of the genera detected. D. Venn diagram of significant genera from differential abundance analysis.

Table 1 Mantel correlations of inter-sample distances between QIIME, mothur and Hybrid-denovo. Bray-Curtis distance matrices on the OTU data are used. *Hybrid-denovo* are run on data sets with different percentages of good quality R2 reads (100%, 75%, 50% and 25%). Top right: Mantel correlation P value based on 1,000 permutation; bottom left: Mantel correlation coefficients.

	Mothur	QIIME	Hybrid(100%)	Hybrid(75%)	Hybrid(50%)	Hybrid(25%)
Mothur	-	< 0.001	< 0.001	< 0.001	< 0.001	< 0.001
QIIME	0.884	-	< 0.001	< 0.001	< 0.001	< 0.001
Hybrid (100%)	0.986	0.879	-	< 0.001	< 0.001	< 0.001
Hybrid (75%)	0.973	0.909	0.985	-	< 0.001	< 0.001
Hybrid (50%)	0.973	0.928	0.982	0.984	-	< 0.001
Hybrid (25%)	0.955	0.949	0.960	0.980	0.985	-

Tables

Additional Files

Additional file 1 — Supplementary Figure 1

Percentage of reads remaining after QC 2013-2015 in Mayo Clinic Sequencing Core Facility

Additional file 2 — Supplementary Figure 2

Hybrid-denovo workflow

Additional file 3 — Supplementary Figure 3

Correlations of 54 prevalent genera (>10%) to the gold standard

Additional file 4 — Supplementary Figure 4

Comparison of DADA2, LotuS and *Hybrid-denovo* on genus-level profiles. All pipelines are run on data sets with 100% good quality R2 reads (“gold standard”). Each column represents the microbiota profile of an individual averaged over all replicates.

Additional file 5 — Supplementary Figure 5

Comparison of DADA2, LotuS and *Hybrid-denovo* on intra-class correlation coefficients (ICCs) of the core genera (A) and OTUs (B). ICCs are calculated based on the technical replicates for six different fecal collection methods. All pipelines are run on data sets with 100% good quality R2 reads (“gold standard”).

Additional file 6 — Supplementary Table 1

Number of reads for the RA dataset after quality control

Additional file 7 — Supplementary Note 1

Details of the steps and parameter settings used for comparing *hybrid-denovo*, QIIME and mothur. Command lines to run the pipelines including DADA2 and LotuS are supplied for transparency.

Availability and requirements
Project name: Hybrid-denovo
Project home page: http://bioinformaticstools.mayo.edu/research/hybrid-denovo/
Operating system(s): Linux (centOS 6 is prefered)
Programming language: Python 2.7, Java and shell script.
Other Requirements: QIIME and python libraries: biom-format (ver 1.3.1), bitarray (ver 0.8.1), pyqi (ver 0.2.0), numpy (ver 1.8.1) and biopython (ver 1.66).
License: Modified BSD.
Any restrictions to use by non-academics: None.
Availability of supporting data
The example files and additional data sets supporting the results of this article are available in the GigaScience Database (http://gigadb.org/), as well as from the project home page.

Figure

Click here to download Figure figure1.pdf

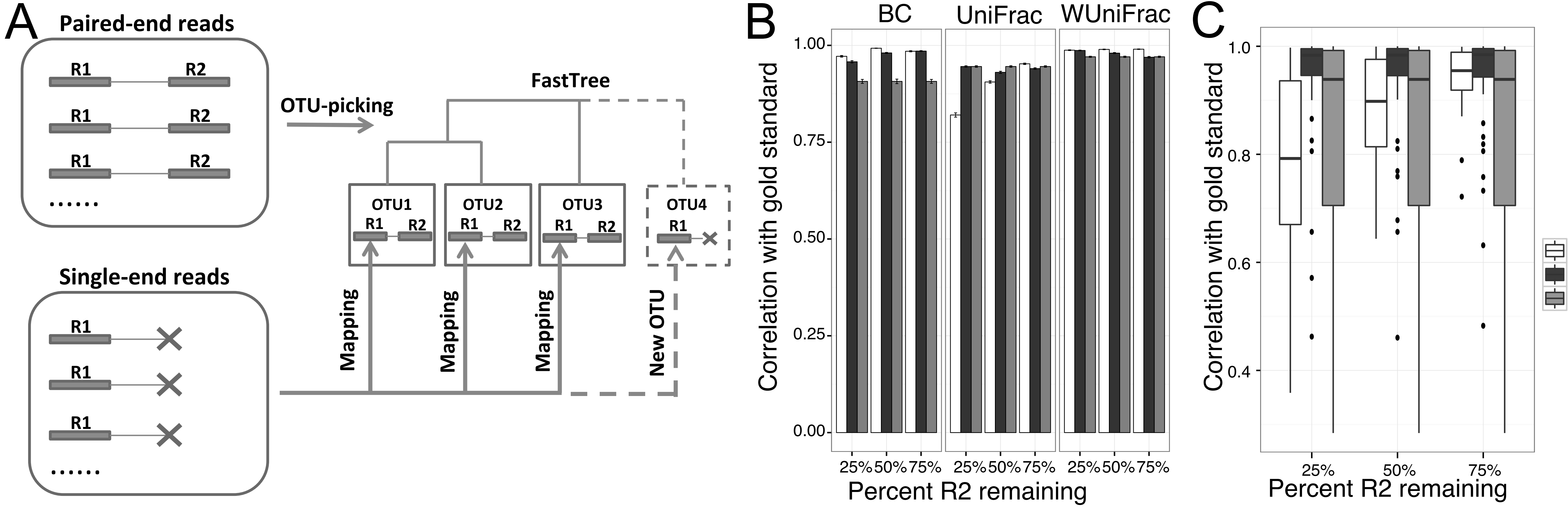

Figure

Click here to download Figure figure2.pdf

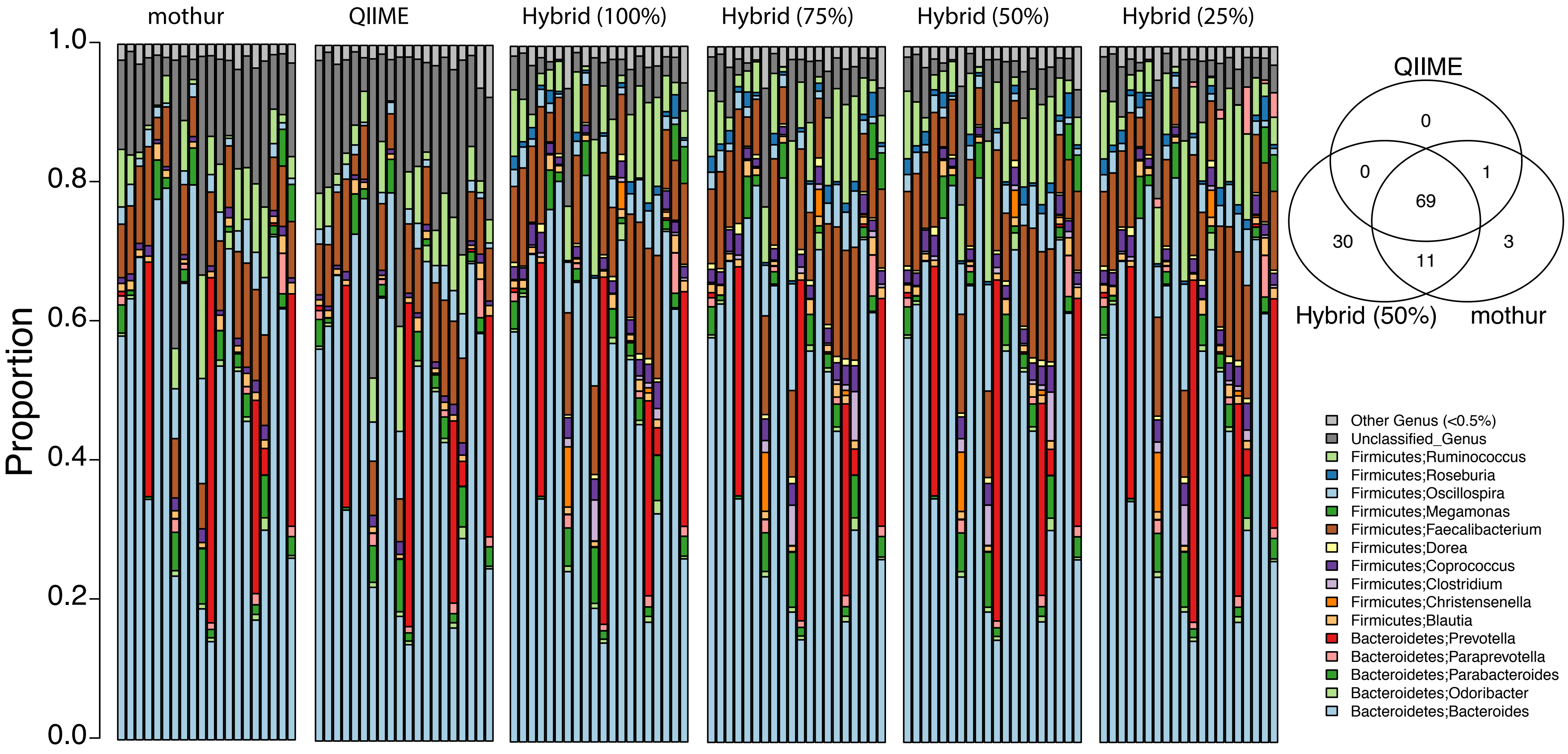

Figure

Click here to download Figure figure3.pdf

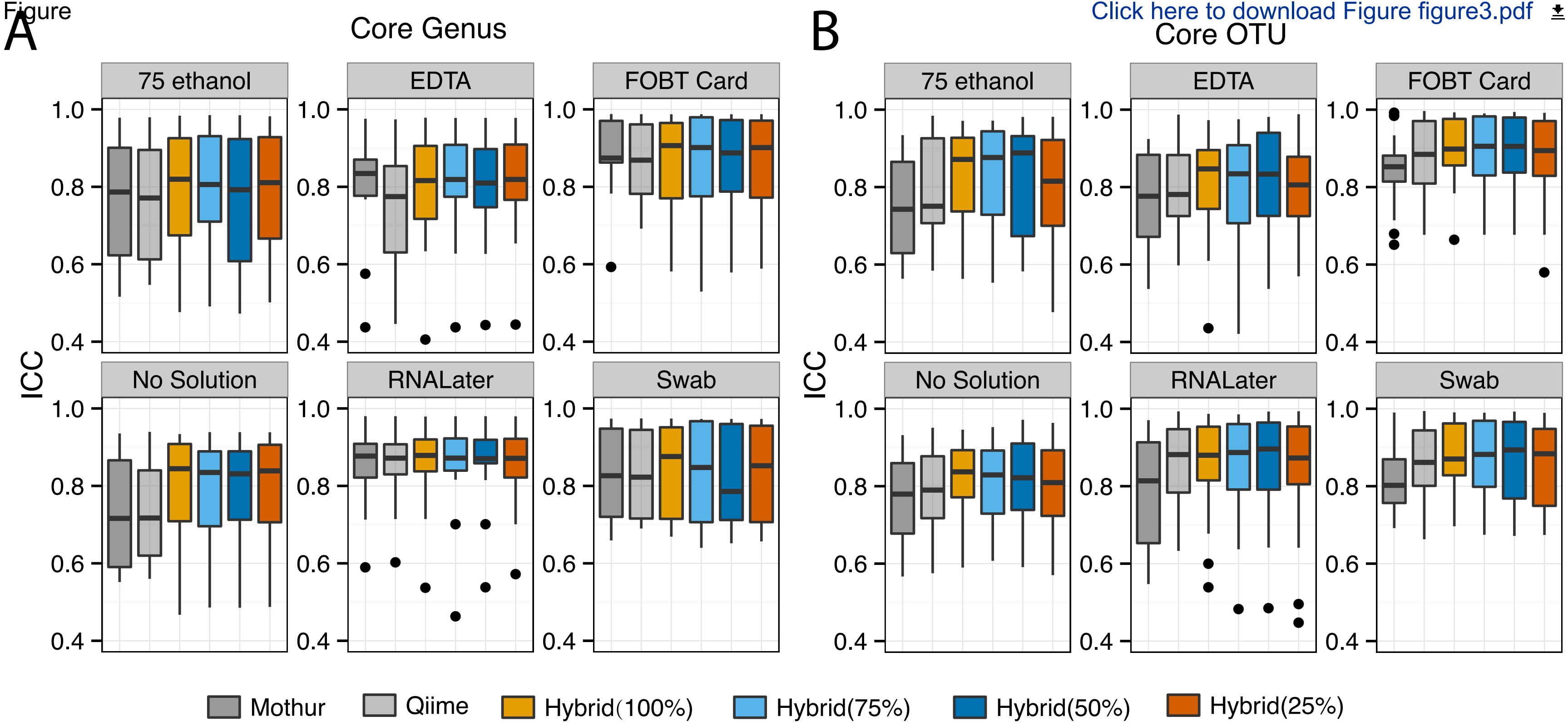

Figure
Click here to download Figure figure4.pdf

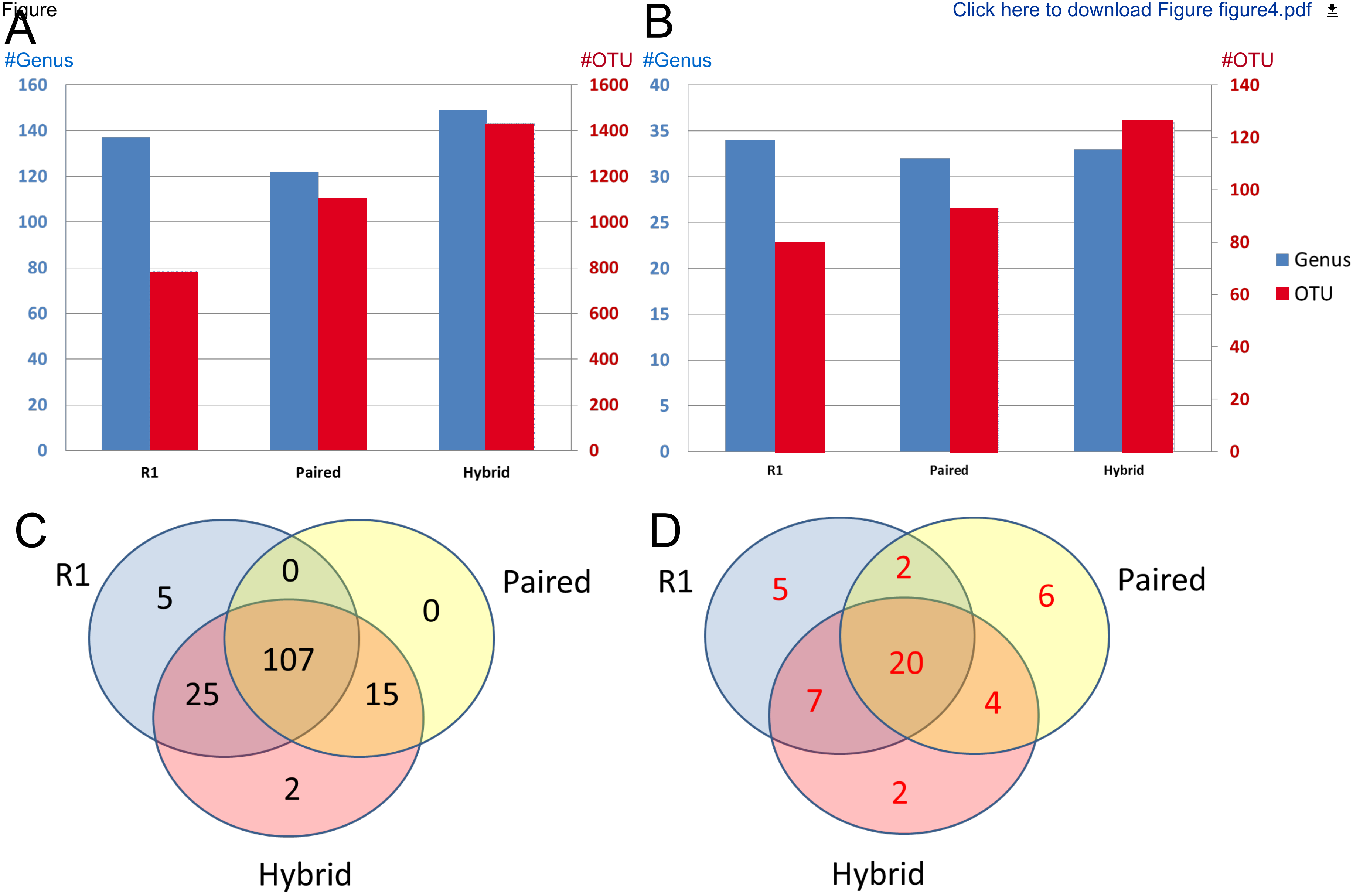

Click here to access/download
Supplementary Material
SupplementaryFigure1.pdf

Click here to access/download
Supplementary Material
SupplementaryFigure2.pdf

Supplementary Material

Click here to access/download
Supplementary Material
SupplementaryFigure3.pdf

Click here to access/download
Supplementary Material
SupplementaryFigure4.pdf

Click here to access/download
Supplementary Material
SupplementaryFigure5.pdf

Click here to access/download

Supplementary Material

SupplementaryNote1.pdf

Supplementary Material

Click here to access/download

Supplementary Material

SupplementaryTable1.png

 Click here to download Personal Cover Response_to_reviewers_comments_JC_2017_08_09_SJ_JC.doc

Dear Editor,

Thanks for giving us the opportunities to resubmit our manuscript (GIGA-S-17-00159). We have seriously considered the comments from you and the reviewer, and made the according revisions. Specifically, in this new revision:

(1) We have added Supplementary Note 1 documenting all the steps used to run the respective pipelines for more transparency and reproducibility.
(2) We have re-run QIIME and mothur with the parameter settings as close as possible to that of hybrid-denovo. For example, we applied the same read quality filter, the same taxonomy classifier (RDP classifiers trained on Greengenes 13.5), and the same taxonomy filter (removed unassigned and non-bacteria OTUs).
(3) We also compared hybrid-denovo to DADA2 and LotuS using the gold standard data set to show the competitive performance of our pipeline (Supplementary Figure 4&5)

With all these changes, the basic conclusion is still the same: increased taxonomic resolution and better reproducibility than QIIME and mothur. Please see the point-to-point response to the reviewer below. We hope you will find this new version satisfactory.

Best,

Jun Chen & Xianfeng Chen

Response to Reviewer #1 comments

In the revision of Chen et al. the authors have made substantial additions to their previous manuscript. However, while it now reads a lot better, a few essential points are still missing and the comparison to state-of-the-art software is simply missing, that would have been more appropriate to get a good idea of the performance of Hybrid-denovo in the year 2017. The software seems to excel at a few things (like taxonomic assignments, but see comments below) but seems to avoid in other parts a fair comparison with software that could get to the same or better levels. Further, I am not convinced that the results might not be driven by false positives. I will recommend this paper for major revision, because I think the high ICC values hold some potential. I fear that the increased taxa assignment rate may be an artifact of a not completely described methodological twist, and that this was not correctly described in the methods. The ICC values I could also imagine to be artifacts due to several factors outlined below. However, I think when the comparisons are done with more comparability and openly described, and if the authors still have higher ICC values than Qiime and mothur, I could imagine this paper to be of scientific value.

In my first review I was not commenting on some of these points; only in the revision I became aware of some potential problems due to better (but still not sufficient) described methods.
Response: Thanks for giving us the opportunities to revise. We have added Supplementary Note 1 documenting all the steps used to run the respective pipelines for more transparency and reproducibility. We re-ran QIIME and mothur with the parameter settings as close as possible to that of hybrid-denovo. For those that could not be the same, we used the methods/parameters suggested by the authors.

Major:
OTU picking / "Gold" Standard: In the methods section, for all used software packages the exact parameters should be mentioned and why these options were used. Especially in a methods paper this is essential for transparency. About the usearch clustering (that is somewhat outdated), here I have a major issue: How do the authors cluster paired end reads with usearch? To the best of my knowledge this is not supported by usearch, so I wonder how this is done within the pipeline? This needs to be described, as simpler approaches like "stitching" will in all likelihood introduce errors.
Response: Thanks for this constructive comment. We added Supplementary Note 1 documenting all the commands used to execute the pipelines for more transparency and reproducibility. The parameter settings were selected to be as close as possible between pipelines. For example, we used Trimmomatic to create good-quality reads, and there is no additional read QC filter for all the pipelines. We used RDP classifiers trained on Greengenes v13.5 to classify reads for all pipelines, and filtered out all singletons and OTUs not belonging to Bacteria.

We used USEARCH with UPARSE-OTU algorithm (https://www.drive5.com/usearch/manual/cmd_cluster_otus.html) for clustering paired-end reads was described in more detail in our previously developed IM-TORNADO pipeline, which was used as the basis for developing our approach (http://journals.plos.org/plosone/article?id=10.1371/journal.pone.0114804). To satisfy the global alignability requirement of UPARSE algorithm, surviving (non-overlapping) read pairs were trimmed down to a specified read length. In our gold standard data set, for the R1, we trimmed down to 250bp; for R2, we trimmed down to 200bp. We then concatenated R1 and R2 together for OTU picking by UPARSE. For taxonomy assignment, we stitched matching reads together with a string consisting of a few ambiguous nucleotides (the character "N") between R1 and R2. A k-mer based approach will ignore k-mers containing ambiguous bases during the classification steps.

We have expanded the method section accordingly. See text highlighted in red in the "Methods" section.

This then goes back to the "Gold" standard: Trying to achieve the same results as the Gold standard assumes that the Gold standard represents some truth; since this is not simulated data where we know the true outcome, but a clinical dataset, this definition of "best" is somewhat problematic since the Gold standard might as well represent the most wrong interpretation of the data. Not surprisingly, the more close the filtered (it is not a simulation, but a read filtering that is done in the 25,50,75% datasets) parameters allow for full (100%) coverage, the more similar the results get to the full ("Gold") standard read set. This is circular reasoning. Further, this introduces a serious theoretical problem: if the Gold standard would be biased to artificial / false positive OTUs of a specific signature, then this would not represent an improved performance but a decrease. Using ICC is a good idea, but can this refute that a false positive bias might drive this benchmark?

Response: Since the paired-end approach on the "gold standard" used all the sequence information from both high-quality R1s and R2s, it is expected to produce higher taxonomic resolution than the single-end approach or paired-end approach on a subset of the read pairs, both of which used only partial sequence information. Therefore, instead of perusing the "truth", we are trying to show that hybrid-denovo approaches has better ability to recover results based on the full sequence information than single-end or paired-end approaches. Thus we believe the results using the "gold standard" are closer to the truth than the results on only R1 reads, everything else being equal. The ICC analysis also showed that the paired-end approach on the "gold standard" (hybrid-deonovo with 100% of R2 reads) usually had a higher ICC value, indicating the results on the "gold standard" had a lower noise level. If the "gold standard" is biased toward false OTUs, we would expect to see a lower ICC value. This is because these false OTUs represent technical noise, and would not appear consistently in replicates.

Artificially increased diversity: the "Discussion" of this important topic now includes a single sentence saying there is a newer software. This still doesn't answer my question, how this software deal with this important problem? I am so insistent on this, since Usearch compared to Uparse usually has increased OTU diversity, and an algorithm like dada2 might even further decrease it. Further the authors mention that they do very stringent read quality filtering. From what I can see this is trimomatic (with what I would regard as lenient parameters, e.g. Q=15 allows for error prone reads to pass) which has considerably less fine grained quality filtering than e.g. Mothur and Qiime, nor the parameter breadth and probabilistic read filtering in the LotuS pipeline, nor the denoising in dada2, nor the abundance corrected clustering of Uparse. Thus this part of the pipeline is in my opinion not state of the art.
Response: For OTU clustering, we used USEARCH with UPARSE-OTU algorithm, it is recommended by author since USEARCH v7. USEARCH is a package and UPARSE is a command option since USEARCH v7. We now revised the discussion and acknowledged the potential limitation of our current implementation. Please see the last paragraph in "Discussion".

Further, it seems like the authors use CMalign to remove reads / OTUs (not sure which) that are not belonging to bacteria. I see two problems with this: 1) This is only mentioned in the Discussion, please expand on this in the Methods section. Also expand the methods to all other steps that are not explicitly mentioned so far.
Response: CMalign is the structure alignment algorithm implemented in "Infernal". We now used "Infernal" instead of "CMalign" to avoid confusion. We also expanded the Methods section to clearly document the major steps. Please see the text highlighted in red.

2) This will for sure bias the dataset, as this is comparable to closed ref OTU picking (only use OTUs/reads that have a representative in the databases). I would also assume that such a treatment will bias the ICC values. This would also explain, why the fraction of assigned reads is higher in Hybrid-denovo (since all OTUs not fitting to bacteria are removed, before taxonomy is compared). Please note somewhere, how many OTUs are actually removed due to the different filtering steps and compare how many reads are in the final OTU matrix between mothur, QIIME and HybridDeNovo.
Response: All the pipelines (Hybrid-denovo, QIIME and mothur) had a similar component to remove non-16S reads (Supplementary Note 1). All non-bacteria OTUs or un-classified OTUs for all pipelines were removed after taxonomic assignments by RDP classifier, allowing for the results from all three pipelines to be compared. The differences of the pipelines are now included in the Supplementary Note 1 with the number of OTUs after each step indicated where applicable.

This is a philosophical question, whether unassigned OTUs should be removed, but the user needs to be made aware that you loose all diversity that is not represented

in the databases, and the benchmarks need to clearly state this difference between the three tested pipelines.
Response: Thanks for the suggestion. We have made clear this point in the text (see Methods, the second last sentence of the first paragraph). We now documented about the major differences between the tested pipelines in the Supplementary Note 1.

I would recommend using public mock communities, WITHOUT any sort of filtering of only known taxa (which would automatically bias the analysis since only known taxa are being used in mock communities). Showing here that the filtering and use of R2 reads can improve OTU clustering, diversity, and taxonomic assignment rates would be a more appropriate test, in my opinion.
Response: Our paper on the IM TORNADO pipeline has demonstrated that using R2 reads improves performance in terms of taxonomy, phylogeny and beta-diversity based on a mock community study.

Comparability to other pipeline: In the abstract the authors claim that "Existing 16S analysis pipeline can either process paired-end or single-end reads, but not a mixture." First, I do not see if Hybrid deNovo could accept an actual mixture of single and paired end reads, but I suspect it can only process a mixture produced within this pipeline from the paired-end input.
Response: We have updated the pipeline. The pipeline could now accept an actual mixture of paired-end and single-end reads.

Second, as pointed out by Pat Schloss in the response, there is a good reason why the second read is not being used, please see the Uparse paper, that explains these reasons with a lot of detail, but there are several papers by now that point out that the second read should NOT be used in the clustering step, as it will likely increase diversity.
Response: This is a rather debatable topic. We believe that the high-quality R2 reads still have values as demonstrated by the IM TORNADO paper. The problem is how to deal with the mixture of (non-overlapping) paired-end and single-end reads. This is the purpose of our proposed approach.

Third, other pipelines are capable of processing a mixture of paired and single end reads (LotuS), even as input, therefore this statement is wrong.
Response: We have modified the statement. See the modified text highlighted in red in the abstract.

Last, since conceptually ideas in Hybrid-denovo and LotuS are very similar (the biggest difference being that LotuS uses the second read for tax assignments, but not for denovo OTU clustering), I would think it more interesting to compare to Dada2 (better OTU resolution) and LotuS (for better tax resolution), which are both in my experience also faster than mothur and QIIME.

Response: We now added a comparison to LotuS and DADA2 on the 'gold standard' data set (See Discussion, the second last paragraph). The purpose here is to see whether our approach was comparable to these start-of-the-art methods. If not, our hybrid-denovo will have less practical value. We presented results on both the genus-level taxonomic profile, the ICC analysis and Mantel correlation (Supplementary Fig. 4&5). We demonstrated a very competitive performance.

Minor:

Greengenes database has last been updated in 2013 and is out of date in this rapidly evolving field, consider using Silva.

Response: Thanks for this good suggestion. For the manuscript, we stick to the Greengenes database for all pipelines for comparability. We will also provide the other options for our pipeline in the future.

Methods: describe with what parameters mothur and Qiime were run.

Response: We described the parameters fully in the Supplementary Note 1.

Wording: "enjoy", "OTUing", .. seem like a colloquial choice of words.

Response: Thanks. We have changed the wording.

Abstract: "Captured more microbial diversity" -> Really? As far as I can see, Hybrid-denovo had less OTUs predicted than either Qiime or mothur.

Response: Due to variation in filtering steps, different pipelines may not be directly comparable. As mentioned in the abstract, , the comparison is between paired-end and single-end approaches assuming everything else being equal.

Abstract: "identified 30% more diessential.." (diessential -> don't know this word).

Response: Sorry for the typo. We corrected it.

Also 30% more than what? I didn't read about any other pipeline being used on this dataset, so this statement if false, if it simply refers to the pruning of the dataset in order to test technical performance. Further, is this really 30% more? Looking at 5, lines 18+, I can also see that all three Hybrid-denovo approaches have 16%,16% and 20% of possible OTUs being classified as sign. different in RA, so the "30%" more could just refer to more OTUs being available, that can be tested for significance?

Response: Yes, the reviewer is right that the three hybrid-denovo approaches detected similar proportions of OTUs as significant. However, we believe, in this example, the absolute number (not proportions) may be more meaningful. Since the hybrid approach use more sequence information, it has higher phylogenetic resolutions. The larger number of significant OTUs/taxa (even the proportion is similar) could reveal more biology due to the increased resolution. For example, we identified more genera, which would be otherwise missed by other methods. We made clear in the abstract that the comparison is between paired-end and single-end approaches

Page 4, line 37+: It is not mentioned, what parameters were used to assign the taxonomy of Qiime and mothur OTUs (RDP classifier maybe?).
Response: We all used RDP classifier trained on Greengenes v13.5. We have made it clear in the method section.

Page 6, line 10+: This is an empty statement and could be easily tested; correctly merging and quality controlling merged reads is not as straightforward as suggested here, a user could probably with less hassle adapt Qiime or mothur to do "hybrid-denovo" assemblies with these pipelines. If you think Hybrid denovo is more powerful than standard pipelines, please demonstrate it.
Response: Thanks for the rigor. We now removed the statement.
