# Supplementary material for: Hybrid-denovo: a de novo OTU-picking pipeline integrating single-end and paired-end 16S sequence tags
Source: Gigascience. 2017 Dec 15;7(3):gix129. doi: 10.1093/gigascience/gix129 (PMC5841375; doi:10.1093/gigascience/gix129)
Supplement: GIGA-D-17-00280_Revision_1.pdf [file gix129_giga-d-17-00280_revision_1.pdf]

GigaScience

Hybrid-denovo: A de novo OTU-picking pipeline integrating single-end and paired-end 16S sequence tags

--Manuscript Draft--

**Manuscript Number:**	GIGA-D-17-00280R1
**Full Title:**	Hybrid-denovo: A de novo OTU-picking pipeline integrating single-end and paired-end 16S sequence tags
**Article Type:**	Technical Note
**Funding Information:**	Center for Individualized Medicine, Mayo Clinic — Dr. Xianfeng Chen
**Abstract:**	Background: Illumina paired-end sequencing has been increasingly popular for 16S rRNA gene-based microbiota profiling. It provides higher phylogenetic resolution than single-end reads due to a longer read length. However, the reverse read (R2) often has significant low base quality and a large proportion of R2s will be discarded after quality control, resulting in a mixture of paired-end and single-end reads. A typical 16S analysis pipeline usually processes either paired-end or single-end reads but not a mixture. Thus, the quantification accuracy and statistical power will be reduced due to the loss of a large amount of reads. As a result, rare taxa may not be detectable with paired-end approach or low taxonomic resolution will be resulted with single-end approach.<br>Findings: To have both the higher phylogenetic resolution provided by paired-end reads and the higher sequence coverage by single-end reads, we propose a novel OTU-picking pipeline, hybrid-denovo, that can process a hybrid of single-end and paired-end reads. Using high quality paired-end reads as a "gold standard", we show that hybrid-denovo achieved the highest correlation with the "gold standard" and performed better than the approaches based on paired-end or single-end reads in terms of quantifying the microbial diversity and taxonomic abundances. By applying our method to a rheumatoid arthritis (RA) data set, we demonstrated that hybrid-denovo captured more microbial diversity and identified more RA-associated taxa than paired-end or single-end approach.<br>Conclusions: Hybrid-denovo utilizes both paired-end and single-end 16S sequencing reads, and is recommended for 16S rRNA gene targeted paired-end sequencing data.
**Corresponding Author:**	Jun Chen<br>Mayo Clinic Rochester<br>Rochester, Minnesota UNITED STATES
**Corresponding Author Secondary Information:**	
**Corresponding Author's Institution:**	Mayo Clinic Rochester
**Corresponding Author's Secondary Institution:**	
**First Author:**	Xianfeng Chen

Powered by Editorial Manager® and ProduXion Manager® from Aries Systems Corporation

**First Author Secondary Information:**	
**Order of Authors:**	Xianfeng Chen
	Stephen Johnson
	Patricio Jeraldo
	Junwen Wang
	Nicholas Chia
	Jean-Pierre A Kocher
	Jun Chen
**Order of Authors Secondary Information:**	
**Response to Reviewers:**	Dear Editor<br><br>Thanks for the acceptance of our manuscript. We have corrected some typos, modified the statement "Hybrid-denovo is more powerful than de novo OTU picking approaches based on paired-end or single-end 16S sequence tags" and uploaded LaTex source files according to your suggestion. We have also deposited the data and code on GigaDB and supplied RRID number in the manuscript.<br><br>Best,<br><br>Jun Chen & Xianfeng Chen
**Additional Information:**	
**Question**	**Response**
Are you submitting this manuscript to a special series or article collection?	No
**Experimental design and statistics**<br><br>Full details of the experimental design and statistical methods used should be given in the Methods section, as detailed in our Minimum Standards Reporting Checklist. Information essential to interpreting the data presented should be made available in the figure legends.<br><br>Have you included all the information requested in your manuscript?	Yes
**Resources**<br><br>A description of all resources used, including antibodies, cell lines, animals and software tools, with enough information to allow them to be uniquely identified, should be included in the Methods section. Authors are strongly encouraged to cite Research Resource Identifiers (RRIDs) for antibodies, model organisms and tools, where possible.	Yes

Have you included the information requested as detailed in our Minimum Standards Reporting Checklist?	
**Availability of data and materials**<br><br>All datasets and code on which the conclusions of the paper rely must be either included in your submission or deposited in publicly available repositories (where available and ethically appropriate), referencing such data using a unique identifier in the references and in the "Availability of Data and Materials" section of your manuscript.<br><br>Have you have met the above requirement as detailed in our Minimum Standards Reporting Checklist?	Yes

Powered by Editorial Manager® and ProduXion Manager® from Aries Systems Corporation

Manuscript

Click here to download Manuscript bmc_article.tex

RESEARCH

Hybrid-denovo: A de novo OTU-picking pipeline integrating single-end and paired-end 16S sequence tags

Xianfeng Chen[1], Stephen Johnson[1], Patricio Jeraldo[2], Junwen Wang[1], Nicholas Chia[2], Jean-Pierre A Kocher[1] and Jun Chen[1]*

*Correspondence: chen.jun2@mayo.edu
[1]Department of Health Sciences Research and Center for Individualized Medicine, Mayo Clinic, 55905 Rochester, USA
Full list of author information is available at the end of the article

Abstract

Background: Illumina paired-end sequencing has been increasingly popular for 16S rRNA gene-based microbiota profiling. It provides higher phylogenetic resolution than single-end reads due to a longer read length. However, the reverse read (R2) often has significant low base quality and a large proportion of R2s will be discarded after quality control, resulting in a mixture of paired-end and single-end reads. A typical 16S analysis pipeline usually processes either paired-end or single-end reads but not a mixture. Thus, the quantification accuracy and statistical power will be reduced due to the loss of a large amount of reads. As a result, rare taxa may not be detectable with paired-end approach or low taxonomic resolution will be resulted with single-end approach.

Findings: To have both the higher phylogenetic resolution provided by paired-end reads and the higher sequence coverage by single-end reads, we propose a novel OTU-picking pipeline, *hybrid-denovo*, that can process a hybrid of single-end and paired-end reads. Using high quality paired-end reads as a "gold standard", we show that *hybrid-denovo* achieved the highest correlation with the "gold standard" and performed better than the approaches based on paired-end or single-end reads in terms of quantifying the microbial diversity and taxonomic abundances. By applying our method to a rheumatoid arthritis (RA) data set, we demonstrated that *hybrid-denovo* captured more microbial diversity and identified more RA-associated taxa than paired-end or single-end approach.

Conclusions: *Hybrid-denovo* utilizes both paired-end and single-end 16S sequencing reads, and is recommended for 16S rRNA gene targeted paired-end sequencing data.

Keywords: microbiome; OTU picking; 16S rRNA

Introduction

The microbiome plays an important role in global ecology, nutrient cycling, and disease [1]. Targeted sequencing of the hypervariable region of the 16S rRNA gene is now routinely used to profile microbiota. Identifying related groups of organisms known as operational taxonomic units or OTUs remains a central part of the analysis of microbiome data. Both *de novo* and reference-based approaches have been proposed for processing 16S rDNA reads - each with complementary strengths and weaknesses. *De novo* OTU-picking naively clusters reads based on sequence similarity. It has the advantages of not requiring any prior knowledge or reference about the target molecule, and produces OTU groupings that are more naturally

aligned to the data. However, *de novo* approaches require comparison of the same gene region. Reference-based approaches can get around this limitation, but rely on a pre-existing set of OTU representatives that may or may not be appropriate for a particular dataset [2].

To perform a *de novo* approach, one of the challenges presented by Illumina paired-end reads is that the reverse read (R2) often has a much lower base quality than the forward read (R1). For the 16S datasets generated at the Mayo Clinic Core Facility, only 24% of R2s passed quality control (QC) between 2013-2015 as opposed to 83% for R1s (Supplementary Fig. 1). We are then left with a smaller set of high-fidelity paired-end reads (R1- R2) and a deeper set of single-end reads (R1). Thus, we would have to choose between the more accurate taxonomic identification using R1-R2 or improved detection of rare taxa using R1[3]. To integrate information from both paired-end and single-end reads, we propose *hybrid-denovo*, a pipeline that combines paired-end and single-end reads in order to retain the advantages of *de novo* OTU-picking while maximizing ability to detect rare taxa.

Methods

Hybrid-denovo first constructs an OTU backbone using only paired-end reads. The remaining single-end reads (R1) are mapped to the OTU backbone, creating new OTUs if unmapped (Fig. 1A). The same quality control and OTU-picking process as implemented in IM-TORNADO is used to build the OTU backbone [3]. Specifically, quality filtering was performed using Trimmomatic [4] with a hard cutoff of PHRED score Q3 for 5' and 3' ends of the reads, trimming of the 3' end with a moving average score of Q15, with a window size of 4 bases, and removing any remaining reads shorter than 75% of the original read length. Reads with any ambiguous base calls were discarded. Surviving read pairs were further trimmed down to specified cutoffs to uniform the length of both reads, then concatenated and sorted by cluster size. Afterwards a *de novo* OTU-picking was conducted via UPARSE algorithm [5, 6]. Though UPARSE algorithm has performed *de novo* chimera removal, we additionally used UCHIME [7] to perform a reference based chimera removal against GOLD database (https://drive5.com/uchime/gold.fa), resulting in a set of high quality OTU representatives. We then mapped the single-end R1s to the R1-end of the OTU representatives using USEARCH (if there are multiple hits with the same score, the most abundant one will be chosen by default). The remaining unmapped R1s were clustered into new OTUs via UPARSE algorithm and added to the list of OTUs generated by the paired-end reads. Thus, the OTU representatives consist of a mixture of single-end and paired-end reads. We then aligned all the OTU representatives using the structure alignment algorithm Infernal trained on the Ribosomal Database Project's (RDP) database [8, 9]. OTU representatives that were not aligned were removed since they hypothetically represented non-bacteria. A phylogenetic tree was built from the aligned OTU representatives using FastTree [10]. FastTree has little penalty on end-gaps, which is favorable when processing a mixture of single-end and paired-end reads. Finally, R1 and R2 reads were stitched together with ambiguous nucleotides (a string of "N"s) in between and then assigned a taxonomy by the RDP classifier [11] trained on the Greengenes database (http://greengenes.secondgenome.com/). OTUs not classified as Bacteria and singleton OTUs were removed as they were presumed contaminants. Note that this

step may have lost diversity that is not represented in the database and is a tradeoff between accuracy and completeness. The complete workflow of our pipeline is given in Supplementary Fig. 2.

To validate our approach, we created a "gold standard" data set with high-quality paired-end reads based on the 837 high-coverage human fecal samples sequenced at the Mayo Core Facility (V3-V5 16S amplicon, 694 nt, 357F/926R primers) [12]. These fecal samples were collected from 20 subjects using 6 different methods (no additive, RNAlater, 70% ethanol, EDTA, dry swab, and fecal occult blood test (FOBT)). The samples were immediately frozen or stored in room temperature for four days to study the stability of the microbiota. Each condition had 2-3 technical replicates to assess the reproducibility. We ran Trimmomatic [4] for quality control and trimmed R1s down to 250bp and R2s down to 200bp to ensure high base quality, resulting in non-overlapping paired-end reads. For each sample, we retrieved 8,000 high-quality paired-end reads. We then performed OTU-picking and taxonomy assignment based on these paired-end reads using IM-TORNADO. These resulting OTUs and their associated taxonomy constitute the "gold standard" dataset. We then subset the "gold standard" with 25%, 50% and 75% of R2 reads remaining. The three sub data sets represented different levels of R2 quality encountered in practice. We compared *hybrid-denovo* to *de novo* approaches based on single-end R1s or paired-end reads using the sub data sets. Performance was evaluated by calculating the Spearman's correlation with the "gold standard" in terms of microbial β-diversity (unweighted and weighted UniFrac, and Bray-Curtis distance) and genus-level relative abundances.

We also compared our pipeline to QIIME and mothur (version 1.8.0 and 1.39.3 respectively) [13, 14] on the "gold standard" data set. Since QIIME and mothur currently do not support *de novo* OTU-picking based on non-overlapping reads, we ran QIIME and mothur on the R1 reads. Parameter settings were chosen to be as comparable to that of *hybrid-denovo*. Since we created good quality-reads by using Trimmomatic, we reduced potential variation in performance between pipelines by not applying additional read QC filters. An RDP classifier trained on Greengenes v13.5 was used to classify reads for all pipelines. Singletons and non-bacteria OTUs (based on taxonomy) were filtered out. The major differences between the three pipelines in addition to the commands used to reproduce the results are documented in Supplementary Note 1. We assessed performance by investigating (1) the number of detected genera and percentage of unclassified reads at the genus level, (2) Mantel correlation using Bray-Curtis matrices, and (3) the intra-class correlation coefficient (ICC) for these core OTUs and genera observed in more than 90% of the samples. ICC is a measure of the correlation between the technical replicates. A high value indicates less measurement error. ICC was calculated using the R ICC package [15].

Finally, we demonstrated the performance of the proposed method on a dataset from the study of the stool microbiome of RA (rheumatoid arthritis) patients, which consists of 40 RA patients and 49 controls (V3-V5 16S amplicon, 694 nt) [16]. We applied DESeq2 to the taxa count data for differential abundance analysis [17] and compared the RA-associated OTUs and genera recovered by different approaches.

Results

The correlation of microbial β-diversity with the "gold standard" was generally high for all the three approaches (Fig. 1B). However, the approach based on single-end R1 tends to have a lower correlation when BC distance was used (the single-end R1 approach was invariant to the number of R2s). The paired-end approach, on the other hand, had a much lower correlation for unweighted UniFrac when only 25% R2s remain. This is due to the fact that unweighted UniFrac captures community membership, which is contributed mainly by rare taxa, and many rare taxa are no longer detectable by the paired-end approach due to loss of reads. In contrast, Hybrid-denovo was very robust and had the best or close to the best correlation with the "gold standard" in both diversity measures. For weighted UniFrac distance, the correlation was similarly high for all the three methods since the weighted UniFrac is most influenced by dominant taxa and all the methods quantify these dominant taxa very well (Fig. 1B).

We next studied the performance of taxonomic profiling of the proposed approach. Based on the 56 genera with prevalence greater than 10%, *hybrid-denovo* had much higher correlation with the "gold standard" across all scenarios considered and its performance was not very sensitive to the percentage of R2 remaining (Fig. 1C). In contrast, the performance of paired-end approach depends strongly on the R2 quality and had much lower correlation when R2 quality was low. The single-end R1 approach was invariant to the number of R2s as expected and performed better than the paired-end approach only when R2 quality was low. Supplementary Fig. 3 showed the individual genus correlations. For the single-end approach, two genera showed zero correlation with the "gold standard" because all of their R1 reads were re-classified at the family level due to their short length (*Lachnobacterium* mapped to *Ruminococcaceae* and *Erwinia* mapped to *Enterobacteriaceae*), indicating the increased phylogenetic resolution using paired-end reads. For the paired-end approach, genera with low-abundance exhibited a lower correlation, indicating the decreased quantification accuracy due to loss of paired-end reads.

We also compared *hybrid-denovo* to mothur and QIIME, the two pre-dominant pipelines for 16S data, based on the "gold standard" data set. Mothur and QIIME took around 24 and 6 hours respectively to complete the analysis of the "gold standard" dataset (n=837), compared to around 1 hour for our pipeline. Mothur and QIIME produced a total of 4,599 and 2,898 non-singleton OTUs respectively while *hybrid-denovo* produced 1,094, 1,086, 1,079 and 1,049 non-singleton OTUs on data sets with different percentages of good quality R2 reads (100%, 75%, 50% and 25%). Though our pipeline resulted in a smaller number of OTUs, we detected a larger number of genera than mothur and QIIME. For example, application of *hybrid-denovo* to the data set with 50% good quality R2 reads yielded a total of 110 genera, compared to 70 and 84 for QIIME and mothur respectively (Fig. 2, upper right, Venn diagram). Using BLAST on the paired-end counterparts of the QIIME and mothur-specific genera (classified based on R1 reads) against the Greengenes database re-assigns many of the reads to other genera. This indicates that those genera were probably misclassified due to shorter reads. Though the genus-level microbiota profiles for the 20 subjects were similar for all the pipelines (Fig. 2), *hybrid-denovo* had a much lower proportion of reads with unknown genus identity

(5%) than mothur and QIIME (14% and 18% respectively). Taken together, these observations demonstrated that *hybrid-denovo* had increased taxonomic resolution due to the use of longer reads. Interestingly, all the pipelines could yield similar inter-sample relationship as measured by Mantel correlation coefficients based on Bray-Curtis distance matrices (Table 1). The availability of technical replicates of the data set allows us to compare different pipelines using intra-class correlation coefficients (ICCs). A high ICC indicates less variability introduced by the bioinformatics pipeline. We calculated the ICCs for different fecal collection methods for the core OTUs and genera, which occurred in more than 90 % of the samples. Our pipeline generally had higher ICCs (less variation between technical replicates) than mothur and QIIME (Fig. 3). In contrast, mothur and QIIME did not perform as well on the core OTUs and genera respectively.

We also applied our method to a dataset from a RA study [16], where about 40% R2s were discarded after quality control (Supplementary Table 1). *Hybrid-denovo* resulted in the largest number of OTUs and genera as expected (Fig. 4A), and covered all genera from paired-end approach and the majority genera from single-end R1 approach (Fig. 4C). Among the five R1-specific genera, *Bacteria Firmicutes Clostridia Clostridiales Clostridiaceae 02d0* and *Bacteria Firmicutes Clostridia Clostridiales Clostridiaceae Sarcina* were re-classified to *Bacteria Firmicutes Clostridia Clostridiales Clostridiaceae Clostridium* when their paired-end counterparts were used, indicating that the R1-specific genera were mis-classified due to their short read length.

Besides the comparison of the detected genera, we also demonstrated the advantage of *hybrid-denovo* in the context of differential abundance analysis using DESeq2 [17]. We excluded OTUs that occurred in less than 10% samples from testing. A total of 758, 578 and 393 OTUs were tested using *hybrid-denovo*, paired and R1 approaches, respectively. Due to higher read counts and increased phylogenetic resolution, *hybrid-denovo* recovered more differential OTUs (Fig. 4B). We identified a total of 126 significant OTUs at an FDR-adjusted P value of 0.01 compared to 93 and 80 OTUs for paired-end and single-end R1 approaches, respectively. Since different methods had their own definition of OTUs and direct comparison of the differential OTUs is difficult, we instead compared the genus identity of the identified OTUs. The differential OTUs identified by *hybrid-denovo* were classified into 33 genera, in comparison to 32 and 34 for the paired-end and single-end R1 approaches (Fig. 4B). There were 20 significant genera shared by all three methods (Fig. 4D), many of which were reported by previous studies [16, 18, 19]. For example, *Bacteroides* is enriched in control samples, while *Collinsella, Eggerthella, Prevotella* and *Clostridium* are enriched in RA samples. Even though the total number of differential genera were similar for all the methods, *hybrid-denovo* identified the most genera (n=11) that were shared by either one of the other two approaches, compared to 6 and 9 for paired-end and single-end R1 approach, indicating that the *hybrid-denovo* approach was able to identify differential genera that were otherwise missed by either paired-end or single-end R1 approach. Furthermore, *hybrid-denovo* had the least number of method-specific genera (n=2) in contract to paired-end (n=6) and R1 single-end (n=5). The method-specific genera might be less reliable due to lack of the support from other methods. For example, R1 approach found *Veillonella* to be enriched in control samples, which is conflict with a previous study

[18]. Interestingly, among the two of the *hybrid-denovo* specific genera, *Klebsiella*, which was enriched in healthy people, was reported by Zhang et al. [19].

Discussion

We proposed *hybrid-denovo* for *de novo* OTU-picking based on paired-end 16S sequence tags. Through simulations and real data examples, we showed that our approach had better performance than single-end or paired-end approach in quantifying the microbial diversity and taxonomic abundance, due to the full use of the information in the paired-end reads.

Based on the size of 16S amplicons and the length of the paired-end reads, we could have overlapping or non-overlapping paired-end reads. For example, sequencing of the V4 region (252 nt, 515F/806R primers) produces overlapping paired-end reads while sequencing of the V3-V5 region (694 nt, F357/R926 primers) results in non-overlapping paired-end reads using Illumina MiSeq (250 bp $\times$2). Since QIIME and mothur currently do not support *de novo* OTU-picking based on non-overlapping paired-end reads, the main advantage of our pipeline lies in the ability to process non-overlapping paired-end reads. However, our pipeline could also be applied to overlapping paired-end reads by using PANDAseq [20] to stitch the paired-end reads together. It is noted that some existing pipelines could also process a mixture of paired-end and single-end reads with different capacities. For example, the recently proposed LotuS pipeline uses good-quality R1 reads to build OTUs, followed by a post-clustering merging of R1 and R2 to increase the accuracy of the taxonomy [21]. However, the OTU-level resolution is still determined by R1 reads.

There are new pipelines that have been developed for 16S data. It is interesting to benchmark *hybrid-denovo* against these state-of-the-art pipelines. We selected DADA2 and LotuS [21, 22] for comparison since they have been demonstrated to have an overall better performance than QIIME and mothur and have been increasingly used by the community. We repeated the same analysis on the “gold standard” data set with complete read pairs. The specific command lines used for DADA2 and LotuS are documented in Supplementary Note 1. DADA2 produced 18,389 sequence variants (SVs) while LotuS produced 472 OTUs. The Mantel correlation on the OTU/SV-level Bray-Curtis distance is high between *hybrid-denovo* and LotuS (ρ=0.93) but moderate between *hybrid-denovo* and DADA2 (ρ=0.71). Interestingly, the Mantel correlation on the genus-level Bray-Curtis distance is high between all methods (ρ>0.97), indicating all methods could produce similar genus-level profiles (Supplementary Fig. 4). Similar ICC analysis demonstrated that all the methods had relatively high ICCs but *hybrid-denovo* had overall the best performance (Supplementary Fig. 5).

One problem for *de novo* OTU-picking is the potential inflated OTU number, which could be due to sources such as sequencing errors, chimera and environmental contaminant [6]. In *hybrid-denovo*, we used various quality filtering criteria to reduce the number of spurious OTUs. For example, we applied Trimmomatic [4] to trim and remove reads with low base quality, removed reads with any ambiguous bases, removed singleton OTUs, used the Infernal package [8] to remove non-structurally aligned OTUs and used reference-based UCHIME as an additional chimera removal process[6]. However, even these filters might fall short of reducing inflated diversity

estimate due to unknown sequencing errors. Improving the diversity estimate from *hybrid-denovo* will be the focus of our future work.

Competing interests
The authors declare that they have no competing interests.

Acknowledgements
This study was supported by Mayo Clinic Center for Individualized Medicine.

Author details
[1]Department of Health Sciences Research and Center for Individualized Medicine, Mayo Clinic, 55905 Rochester, USA. [2]Department of Surgery, Mayo Clinic, 55905 Rochester, USA.

References
1. Cho I and Blaser MJ, **The human microbiome: at the interface of health and disease**. *Nat. rev. genet.* 2012;13(4):260-70.
2. McDonald D, Birmingham A, and Knight R. **Context and the human microbiome**. *Microbiome* 2015; 3:52.
3. Jeraldo P, Kalari K, Chen X, Bhavsar J, Mangalam A, White B, et al. **IM-TORNADO: A tool for comparison of 16s reads from paired-end libraries**. *PloS ONE* 2014; 9(12):e114804.
4. Bolger MA, Lohse M, and Usadel B. **Trimmomatic: a flexible trimmer for illumina sequence data**. *Bioinformatics* 2014; 30(15):2114-20.
5. Edgar RC. **Search and clustering orders of magnitude faster than blast**. *Bioinformatics* 2010; 26(19):2460-1.
6. Edgar RC. **Uparse: highly accurate OTU sequences from microbial amplicon reads**. *Nat. methods* 2013;10(10):996-8.
7. Edgar RC, Haas BJ, Clemente JC, Quince C, Knight R. **UCHIME improves sensitivity and speed of chimera detection**. *Bioinformatics* 2011;10(10):27(16):2194-200.
8. Nawrocki EP, Kolbe DL, and Eddy SR. Infernal 1.0: inference of RNA alignments. *Bioinformatics* 2009; 25(10):1335-7.
9. Cole JR, Wang Q, Cardenas E, Fish J, Chai B, Farris RJ, et al. **The ribosomal database project: improved alignments and new tools for rRNA analysis**. *Nucleic Acids Res.* 2009;37:D141?D145.
10. Price MN, Dehal PS, and Arkin AP. **Fasttree 2–approximately maximum-likelihood trees for large alignments**. *PloS ONE* 2010; 5(3):e9490.
11. Wang Q, Garrity GM, Tiedje JM and Cole JR. **Naive Bayesian classifier for rapid assignment of rRNA sequences into the new bacterial taxonomy**. *Appl. environ. microbiol.* 2007; 73(16):5261-5267
12. Sinha R, Chen J, Amir A, Vogtmann E, Shi J, Inman KS, et al. **Collecting fecal samples for microbiome analyses in epidemiology studies**. *Cancer epidemiol. biomarkers prev.* 2016; 25(2):407-16.
13. Caporaso JG, Kuczynski J, Stombaugh J, Bittinger K, Bushman FD, Costello EK, et al. **QIIME allows analysis of high-throughput community sequencing data**. *Nat. methods* 2010;7:335-336
14. Schloss PD, Westcott SL, Ryabin T, Hall JR, Hartmann M, Hollister EB, et al. **Introducing mothur: open-source, platform-independent, community-supported software for describing and comparing microbial communities**. *Appl. environ. microbiol.* 2009; 75(23):7537-41.
15. Wolak ME, Fairbairn DJ, Paulsen YR **Guidelines for Estimating Repeatability** *Methods in Ecology and Evolution* 2012; 3(1):129-137
16. Chen J, Wright K, Davis JM, Jeraldo P, Marietta EV, Murray J, et al. **An expansion of rare lineage intestinal microbes characterizes rheumatoid arthritis**. *Genome med.* 2016; 8(1):43
17. Love MI, Huber W, Anders S. **Moderated estimation of fold change and dispersion for RNA-seq data with DESeq2**. *Genome biol.* 2014; 15(12):550
18. Scher JU, Sczesnak A, Longman RS, Segata N, Ubeda C, Bielski C, et al. **Expansion of intestinal Prevotella copri correlates with enhanced susceptibility to arthritis**. *Elife* 2013; 5(2):e01202
19. Zhang X, Zhang D, Jia H, Feng Q, Wang D, Liang D, et al. **The oral and gut microbiomes are perturbed in rheumatoid arthritis and partly normalized after treatment**. *Nat. med.*, 2015, Aug;21(8):895-905
20. Masella AP, Bartram AK, Truszkowski JM, Brown DG, and Neufeld JD. **Pandaseq: paired-end assembler for illumina sequences**. *BMC bioinformatics* 2012; 13:31
21. Hildebrand F, Tadeo R, Voigt AY, Bork P, Raes J. **LotuS: an efficient and user-friendly OTU processing pipeline**. *Microbiome* 2014; 2:30
22. Callahan BJ, McMurdie PJ, Rosen MJ, Han AW, Johnson AJ, Holmes SP. **DADA2: high-resolution sample inference from Illumina amplicon data**. *Nat. methods* 2016;13:581-3
23. Chen X, Johnson S, Jeraldo P, Wang J, Chia N, Kocher JA, Chen J. **Supporting data for "Hybrid-denovo: A de novo OTU-picking pipeline integrating single-end and paired-end 16S sequence tags"** . *GigaScience Database* 2017;http://dx.doi.org/10.5524/100388

Figures
Tables
Additional Files
Additional file 1 — Supplementary Figure 1
Percentage of reads remaining after QC 2013-2015 in Mayo Clinic Sequencing Core Facility

Additional file 2 — Supplementary Figure 2
Hybrid-denovo workflow

Figure 1 Overview and evaluation of the Hybrid-denovo approach. A. *Hybrid-denovo* illustration. B. Mantel correlation of β-diversity distance matrices (Unweighted UniFrac, Weighted UniFrac and Bray-Curtis distance) with the "gold standard" for the three approaches at different percentages of good-quality R2 reads. Error bars represent standard errors of the estimate based on 100 bootstrap samples. C. Boxplot of correlations of the relative abundances of 56 prevalent genera with the "gold standard".

Figure 2 Comparison of mothur, QIIME and Hybrid-denovo on genus-level profiles. *Hybrid-denovo* are run on data sets with different percentages of good quality R2 reads (100%, 75%, 50% and 25%). Each column represents the microbiota profile of an individual averaged over all replicates. The overlaps of detected genera between the three pipelines are shown in the Venn diagram.

Figure 3 Comparison of mothur, QIIME and Hybrid-denovo on intra-class correlation coefficients (ICCs) of the core genera (A) and OTUs (B). ICCs are calculated based on the technical replicates for six different fecal collection methods. *Hybrid-denovo* are run on data sets with different percentages of good quality R2 reads (100%, 75%, 50% and 25%).

Figure 4 Comparison of the R1, Paired and Hybrid approaches on the RA dataset. A. Number of detected OTUs (red) and genera (blue). B. Number of significant OTUs (red) and genera (blue) from differential abundance analysis (FDR≤ 0.01). C. Venn diagram of the genera detected. D. Venn diagram of significant genera from differential abundance analysis.

Table 1 Mantel correlations of inter-sample distances between QIIME, mothur and Hybrid-denovo. Bray-Curtis distance matrices on the OTU data are used. *Hybrid-denovo* are run on data sets with different percentages of good quality R2 reads (100%, 75%, 50% and 25%). Top right: Mantel correlation P value based on 1,000 permutation; bottom left: Mantel correlation coefficients.

	Mothur	QIIME	Hybrid(100%)	Hybrid(75%)	Hybrid(50%)	Hybrid(25%)
Mothur	-	< 0.001	< 0.001	< 0.001	< 0.001	< 0.001
QIIME	0.884	-	< 0.001	< 0.001	< 0.001	< 0.001
Hybrid (100%)	0.986	0.879	-	< 0.001	< 0.001	< 0.001
Hybrid (75%)	0.973	0.909	0.985	-	< 0.001	< 0.001
Hybrid (50%)	0.973	0.928	0.982	0.984	-	< 0.001
Hybrid (25%)	0.955	0.949	0.960	0.980	0.985	-

Additional file 3 — Supplementary Figure 3
Correlations of 54 prevalent genera (>10%) to the gold standard

Additional file 4 — Supplementary Figure 4
Comparison of DADA2, LotuS and *Hybrid-denovo* on genus-level profiles. All pipelines are run on data sets with 100% good quality R2 reads ("gold standard"). Each column represents the microbiota profile of an individual averaged over all replicates.

Additional file 5 — Supplementary Figure 5
Comparison of DADA2, LotuS and *Hybrid-denovo* on intra-class correlation coefficients (ICCs) of the core genera (A) and OTUs (B). ICCs are calculated based on the technical replicates for six different fecal collection methods. All pipelines are run on data sets with 100% good quality R2 reads ("gold standard").

Additional file 6 — Supplementary Table 1
Number of reads for the RA dataset after quality control

Additional file 7 — Supplementary Note 1
Details of the steps and parameter settings used for comparing *hybrid-denovo*, QIIME and mothur. Command lines to run the pipelines including DADA2 and LotuS are supplied for transparency.

Availability and requirements
Project name: Hybrid-denovo (https://scicrunch.org/SCR_015866)
Project home page: http://bioinformaticstools.mayo.edu/research/hybrid-denovo/
Operating system(s): Linux (centOS 6 is prefered)

Programming language: Python 2.7, Java and shell script.
Other Requirements: QIIME and python libraries: biom-format (ver 1.3.1), bitarray (ver 0.8.1), pyqi (ver 0.2.0), numpy (ver 1.8.1) and biopython (ver 1.66).
License: Modified BSD.
Any restrictions to use by non-academics: None.

Availability of supporting data

The example files and additional data sets supporting the results of this article are available in the GigaScience Database[23], as well as from the project home page.

Figure

Click here to download Figure figure1.pdf

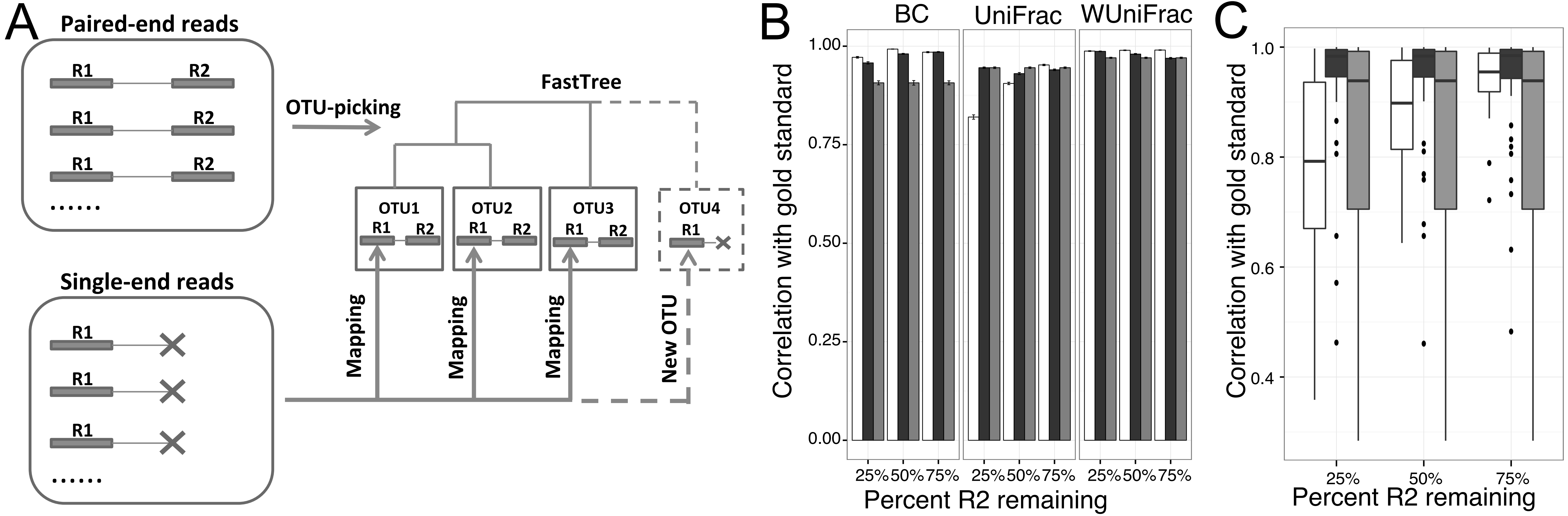

Figure

Click here to download Figure figure2.pdf

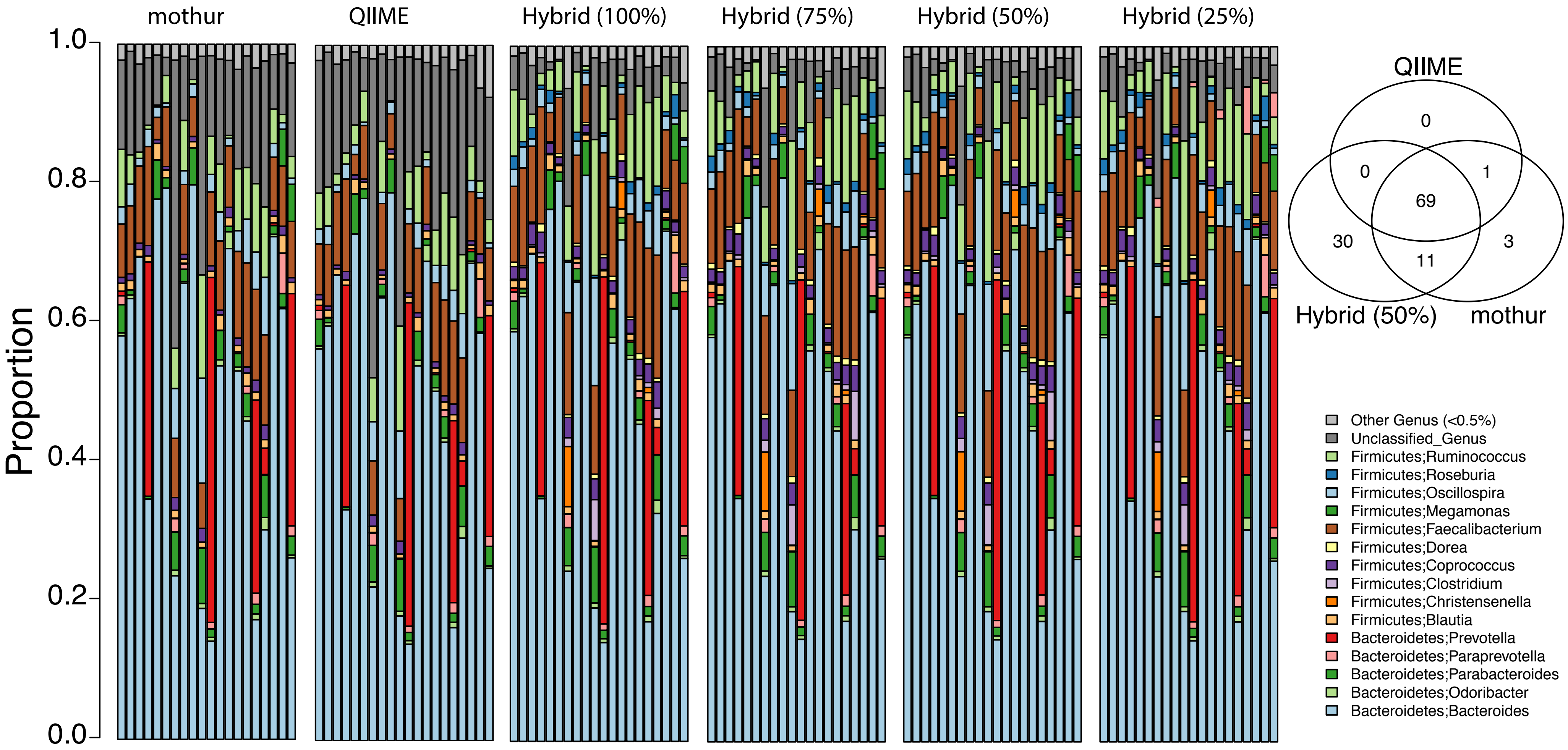

Figure

Click here to download Figure figure3.pdf

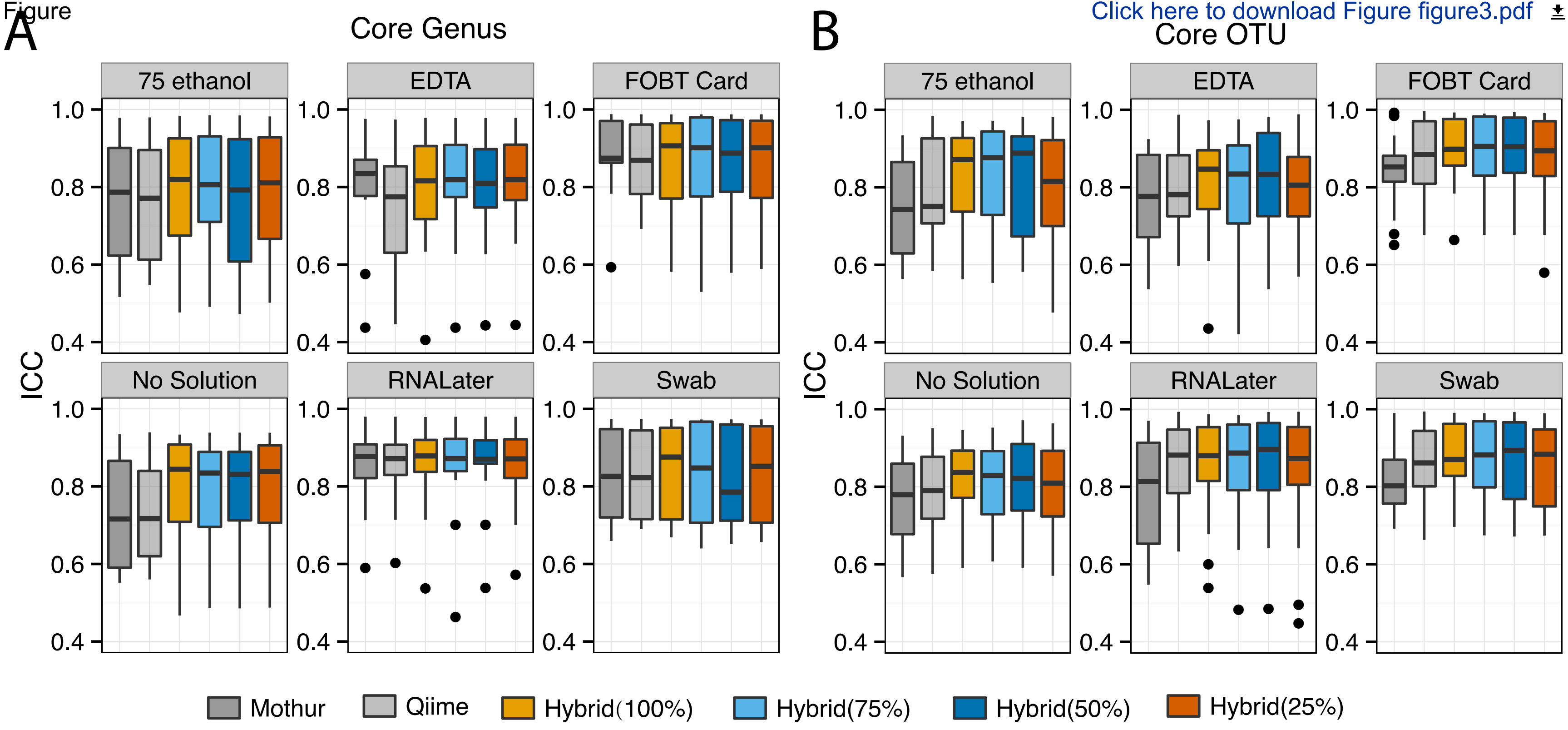

Figure

Click here to download Figure figure4.pdf

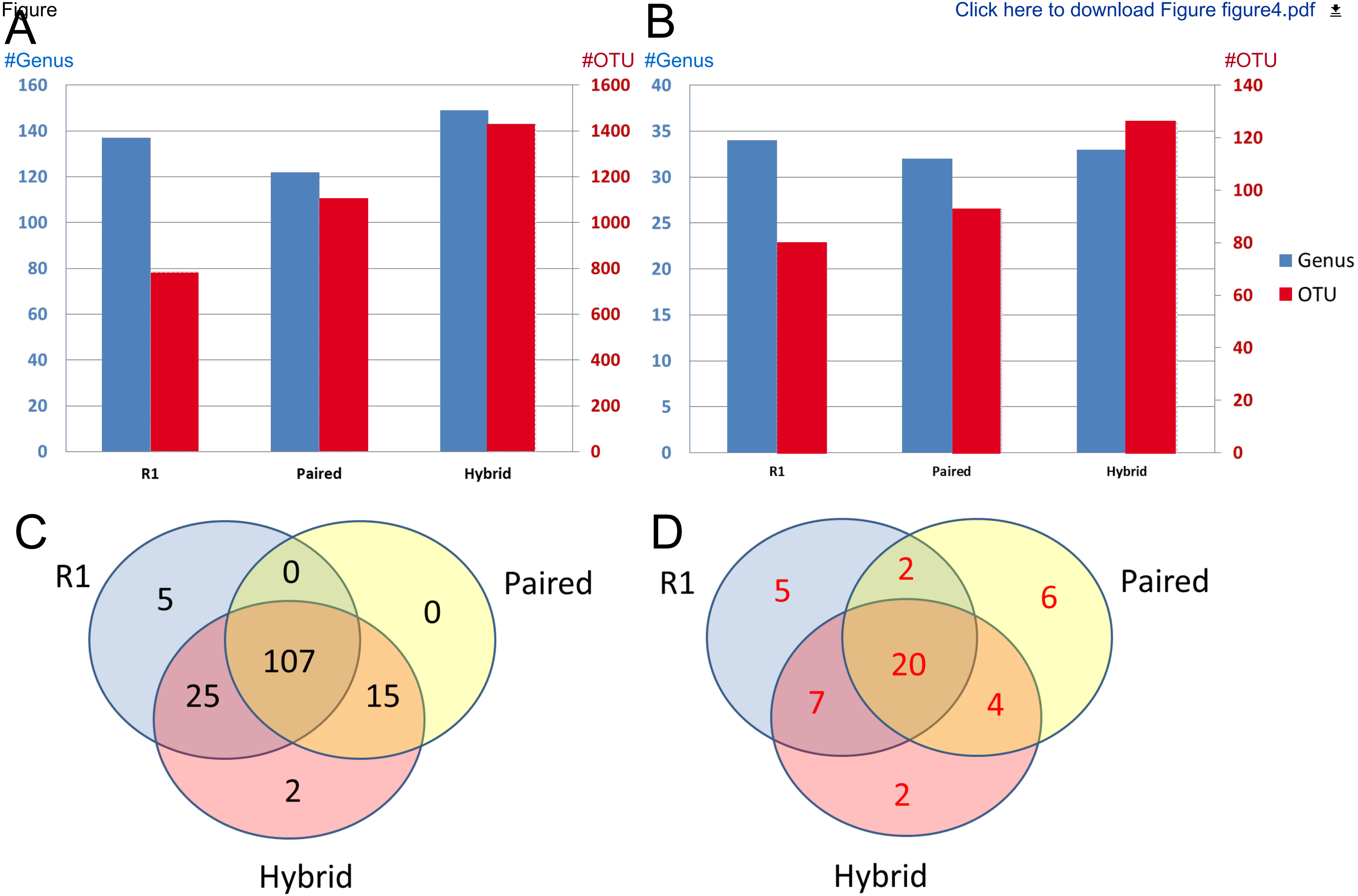

Click here to access/download
Supplementary Material
SupplementaryFigure1.pdf

Click here to access/download
Supplementary Material
SupplementaryFigure2.pdf

Click here to access/download
Supplementary Material
SupplementaryFigure3.pdf

Click here to access/download
Supplementary Material
SupplementaryFigure4.pdf

Click here to access/download
Supplementary Material
SupplementaryFigure5.pdf

Click here to access/download
Supplementary Material
SupplementaryNote1.pdf

Click here to access/download
Supplementary Material
SupplementaryTable1.png
