# Supplementary material for: Hybrid-denovo: a de novo OTU-picking pipeline integrating single-end and paired-end 16S sequence tags
Source: Gigascience. 2017 Dec 15;7(3):gix129. doi: 10.1093/gigascience/gix129 (PMC5841375; doi:10.1093/gigascience/gix129)
Supplement: Supplemental material [file gix129_supp.zip › SupplementaryFigure2.pdf]

Supplementary figure 2

Hybrid-denovo Work Flow

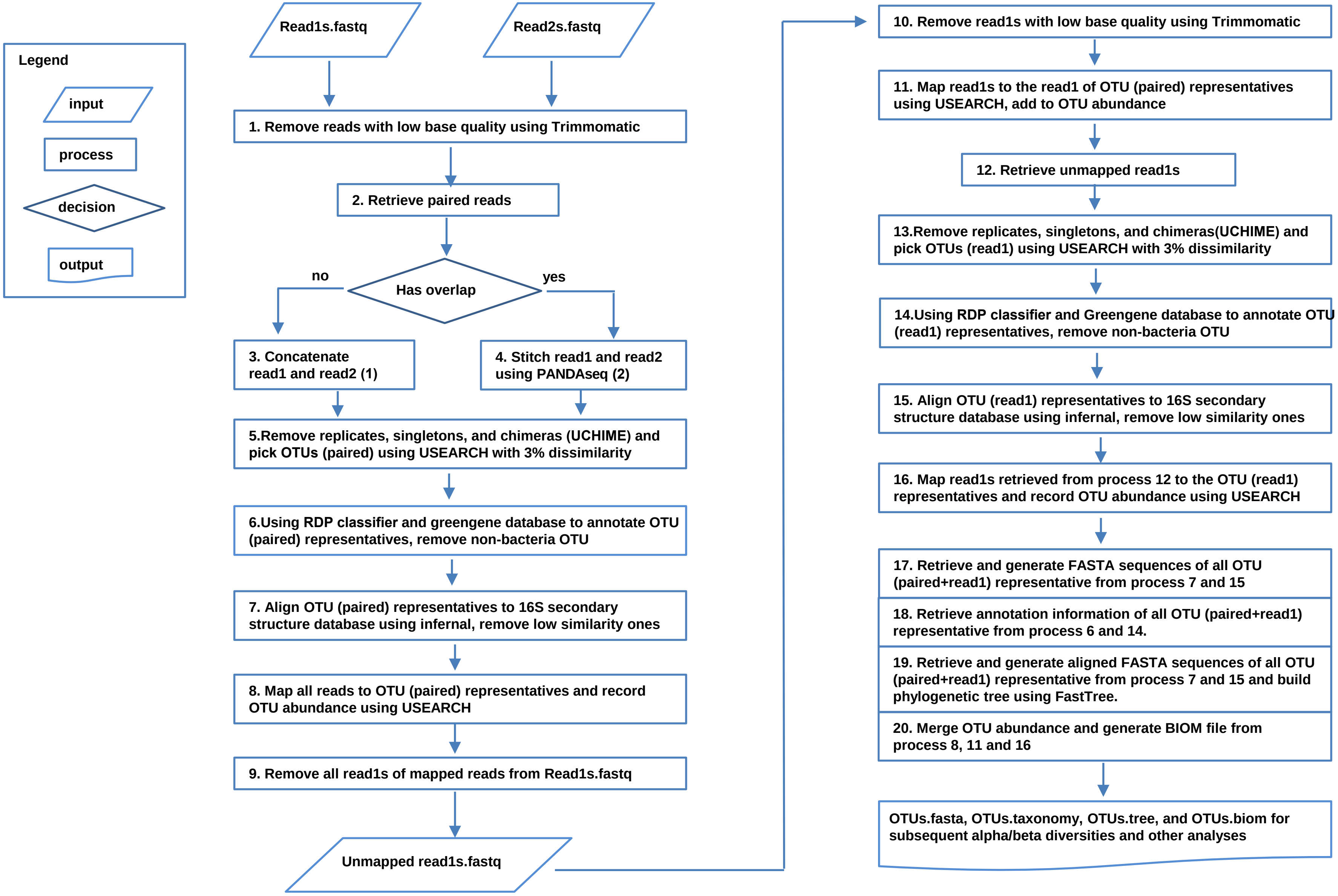

1) Jeraldo, P., et al. (2014) IM-TORNADO: A tool for comparison of 16s reads from paired-end libraries. PloS one, 9(12):e114804.
2) Masella, A.P., *et al.* (2012) PANDAseq: paired-end assembler for illumina sequences, *BMC Bioinformatics*, **13**, 31.
