# Supplementary material for: Hybrid-denovo: a de novo OTU-picking pipeline integrating single-end and paired-end 16S sequence tags
Source: Gigascience. 2017 Dec 15;7(3):gix129. doi: 10.1093/gigascience/gix129 (PMC5841375; doi:10.1093/gigascience/gix129)
Supplement: Supplemental material [file gix129_supp.zip › SupplementaryNote1.pdf]

Workflow Comparisons

- We compared hybrid-denovo with QIIME and mothur pipelines. For each step, we listed pseudo code with important parameters indicated, and we also indicated the number of OTUs (#OTUs) from each step as possible.
- We first filtered our data using Trimmomatic before applying all the methods to eliminate the pipeline-specific QC bias, and generated gold standard dataset for all comparisons.
- For QIIME, we used the same procedures as hybrid-denovo does.
- For mothur, we used the SOP workflow for Illumina recommended by mothur (https://www.mothur.org/wiki/MiSeq_SOP), and we used the same procedures as hybrid-denovo does if applicable.
- We also included the command lines for running LotuS and DADA2 pipelines using default parameter settings, where OTU taxonomies were picked up against Greengenes v13.5

Workflow Comparisons

	Hybrid-denovo	QIIME	mothur
Dataset	Generate gold standard using Trimmomatic for reads quality control. Total paired reads=6,696,000		
Input	100%R1+50%R2<br>#paired reads=3,348,000<br>#remained R1=3,348,000	100%R1+0%R2<br>#reads=6,696,000	100%R1+0%R2<br>#reads=6,696,000
Reads quality control	No	No	No
1) OTU picking	USEARCH<br>#paired_OTU=585<br>#R1_OTU=534 (Unmapped R1=554,729)	USEARCH<br>#Enumerated OTU=3,168	Opticlust<br>#OTUs= 10,104
2) Non-16S reads removal	Infernal<br>#paired_OTU=584<br>#R1_OTU=495	Infernal	Map to SLIVA<br>#unique read=380,020
3) Chimera removal	Reference based UCHIME	Reference based UCHIME	Vsearch<br>#read cluster=103,530
4) Assign Taxonomy and remove non-bacteria OTUs	Method=RDP classifier<br>Database=Greengenes v13.5<br>#paired_OTU=584<br>#R1_OTU=495<br>Total OTU=1,079	Method=RDP classifier<br>Database=Greengenes v13.5<br>#OTU=2,902	Method=RDP classifier<br>Database=Greengenes v13.5<br>#bacteria read cluster =103,515
5) Remove singletons	#OTU=1,079	#OTU=2,898	#OTU=4,599
Process order (steps in parathsis are processed in one module)	(3,1),4,2,5	(3,2,1),4,5	2,3,4,1,5

Generate Gold Standard samples

16 amplicon non-overlap paired reads (Illumina)

↓

Trimmomatic R1: LEADING:3 TRAILING:3 SLIDINGWINDOW:4:15 MINLEN:250
Trimmomatic R2: LEADING:3 TRAILING:3 SLIDINGWINDOW:4:15 MINLEN:200

↓

trim R1=250bp, R2=200bp

↓

Removed un-paired reads

↓

Gold Standard (N=837, 8000 paired reads per sample)

QIIME pipeline(1.8.0)

Gold Standard (R1, read#=6696000)

pick_otus.py -i qiime_R1.fasta -m usearch --db_filepath=gold.fa -o usearch_qf_results/ --word_length 64
denovo clustering using usearch
reference (gold database) based chimeras removal using usearch (enumerated OTU#=3168)

align_seqs.py -i usearch_qf_results/enumerated_otus.fasta -o usearch_qf_results/alignment -m infernal -t seed.16s.reference_model.sto
Align OTUs to 16s database with Infernal (aligned enumerated OTU#=3168)

make_phylogeny.py -i usearch_qf_results/alignment/enumerated_otus_aligned.fasta -o usearch_qf_results/qiime_R1.tree -t fasttree
Build up phylogenetic tree by fasttree

assign_taxonomy.py -i usearch_qf_results/enumerated_otus.fasta -r greengene.fna -t greengene.taxonomy -o usearch_qf_results/taxonomy –m rdp
Assign taxonomy with RDP method against greengenes (13_5-release)

make_otu_table.py -i usearch_qf_results/qiime_R1_otus.txt -o usearch_qf_results/qiime_R1_otus.biom -t usearch_qf_results/taxonomy/enumerated_otus_tax_assignments.txt(OTU#=3157)
filter_taxa_from_otu_table.py -i usearch_qf_results/qiime_R1_otus.biom -o otu_table_bac_only.biom -p k__Bacteria
Generate BIOM file and filter taxonomy by Bacteria (OTU#=2898)

mothur pipeline (based on Illumina SOP)

Gold Standard (R1, read#=6,696,000)

Create virtual fastq of R2s based on R1s (as this SOP is for overlapped paired reads).
1) For fasta sequence, make reverse complete sequence of R1 fasta
2) For quality score, make reverse sequence of R1 quality score

make.file(..,)
make.contigs(..,)
screen.seqs(..,maxambig=0, maxlength=280)
unique.seqs(..,)
count.seqs(..,)
Process overlapped paired reads, remove reads having ambiguous base (read#= 6628400, unique read#= 526,925)

align.seqs(.., reference=silva.fasta
screen.seqs(.., start=1,428, end=1,1307, maxhomop=8)
filter.seqs(.., vertical=T, trump=.)
Remove reads not mapped to silva (read#= 433,920)

unique.seqs(..,)
unique reads again (unique read#=380,020)

mothur pipeline (cont.)

```
pre.cluster(.., diffs=2)
chimera.vsearch(.., dereplicate=t)
remove.seqs(..,)
### Remove chimeras with denovo method (unique read#=103,530)
```

```
classify.seqs(.., reference=greengene.fna, taxonomy=greengene.taxonomy, cutoff=80)
remove.lineage(..,taxon=Chloroplast-Mitochondria-unknown-Archaea)
### map reads to greengene database with RDP classifiying, remove those not belong to “bacteria”
```

```
dist.seqs(.., cutoff=0.03)
cluster(..,)
cluster.split(.., splitmethod=classify, taxlevel=4, cutoff=0.03)
### generate distance matrix, clustering, assign each read to OTU
```

```
make.shared(.., label=0.03)
make.biom(..,)
### Create BIOM file, with 3% dissimilarity (#OTUs= 10,104)
```

```
get.oturep(..,)
dist.seqs(..,cutoff=0.03, output=lt)
clearcut(..,)
### Create distance matrix between OTU representatives and build up their phylogenetic tree
### filter OTUs with abundance >1 (#OTU=4599)
```

LotuS pipeline

Gold Standard (paired, read#=3348000, R1, read#= 3348000)

```
lotus.pl
-I gold_standard_reads
-o output_GG13.5
-m mapping.txt
-s sdm_miSeq.txt
-refdb GG
-simBasedTaxo 0
-p miSeq
```

DADA2 pipeline

Gold Standard (paired, read#=3348000, R1, read#= 3348000)

```
fastqPairedFilter
truncLen = c(250,200)
maxN=0
maxEE=2.0
truncQ= 2
compress=T
verbose=T
```

```
Dada
derep.R1[seq(1,length(seq_along(filt.R1)),30)],
                    err= inflateErr(tperr1,3), selfConsist = T)
 derep.R2[seq(1,length(seq_along(filt.R2)),30)],
                    err= inflateErr(tperr1,3), selfConsist = T)
```

```
mergePairs
dada2.R1
derep.R1
dada2.R2
derep.R2
verbose = T
justConcatenate = T
```

makeSequenceTable

removeBimeraDenovo
