# Supplementary figures and images for: Hybrid-denovo: a de novo OTU-picking pipeline integrating single-end and paired-end 16S sequence tags
Source: Gigascience. 2017 Dec 15;7(3):gix129. doi: 10.1093/gigascience/gix129 (PMC5841375; doi:10.1093/gigascience/gix129)

Supplementary figure 1

## Remaining % reads after QC 2013-2015 in Mayo

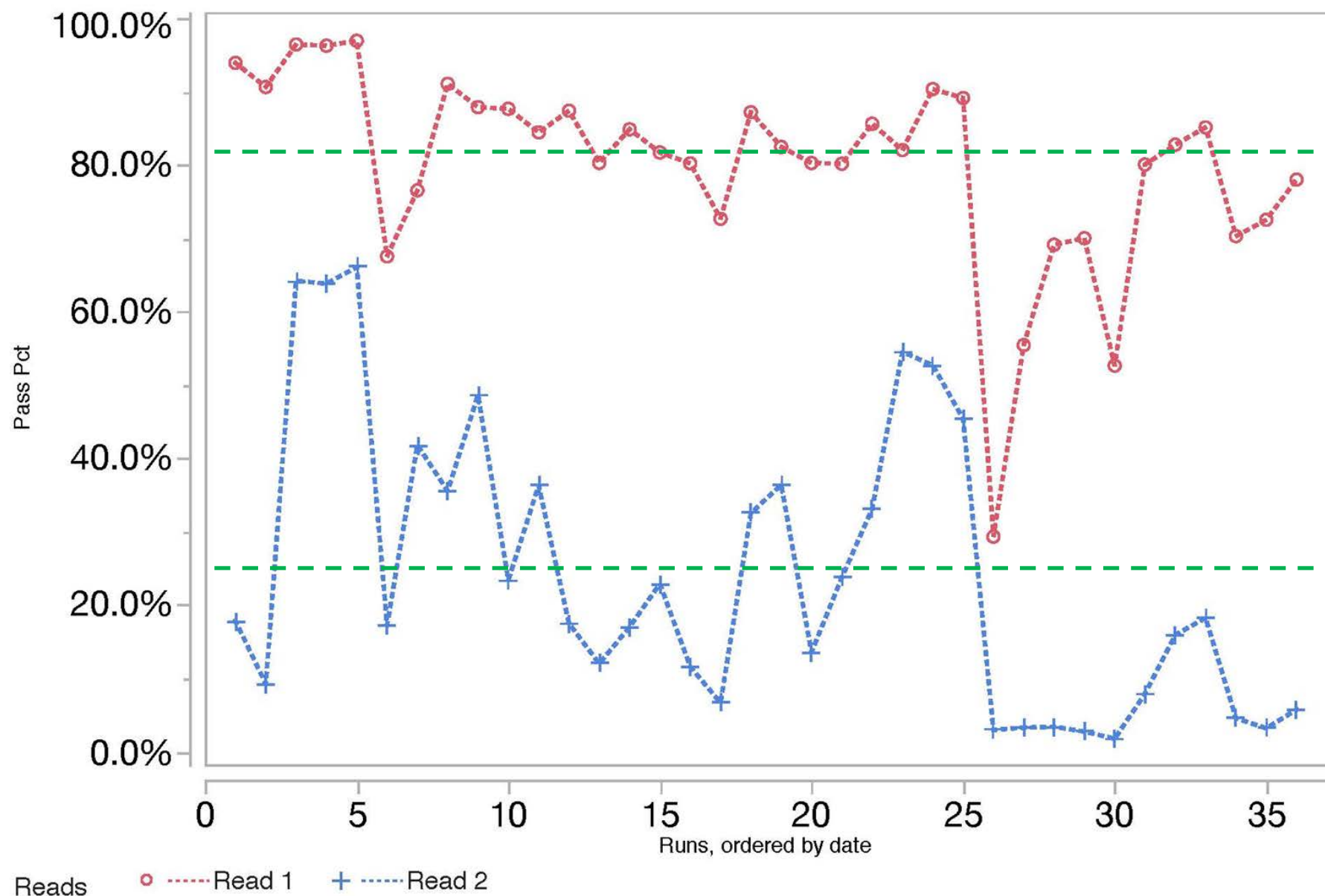

Supplement: Supplemental material [file gix129_supp.zip › SupplementaryFigure1.pdf]

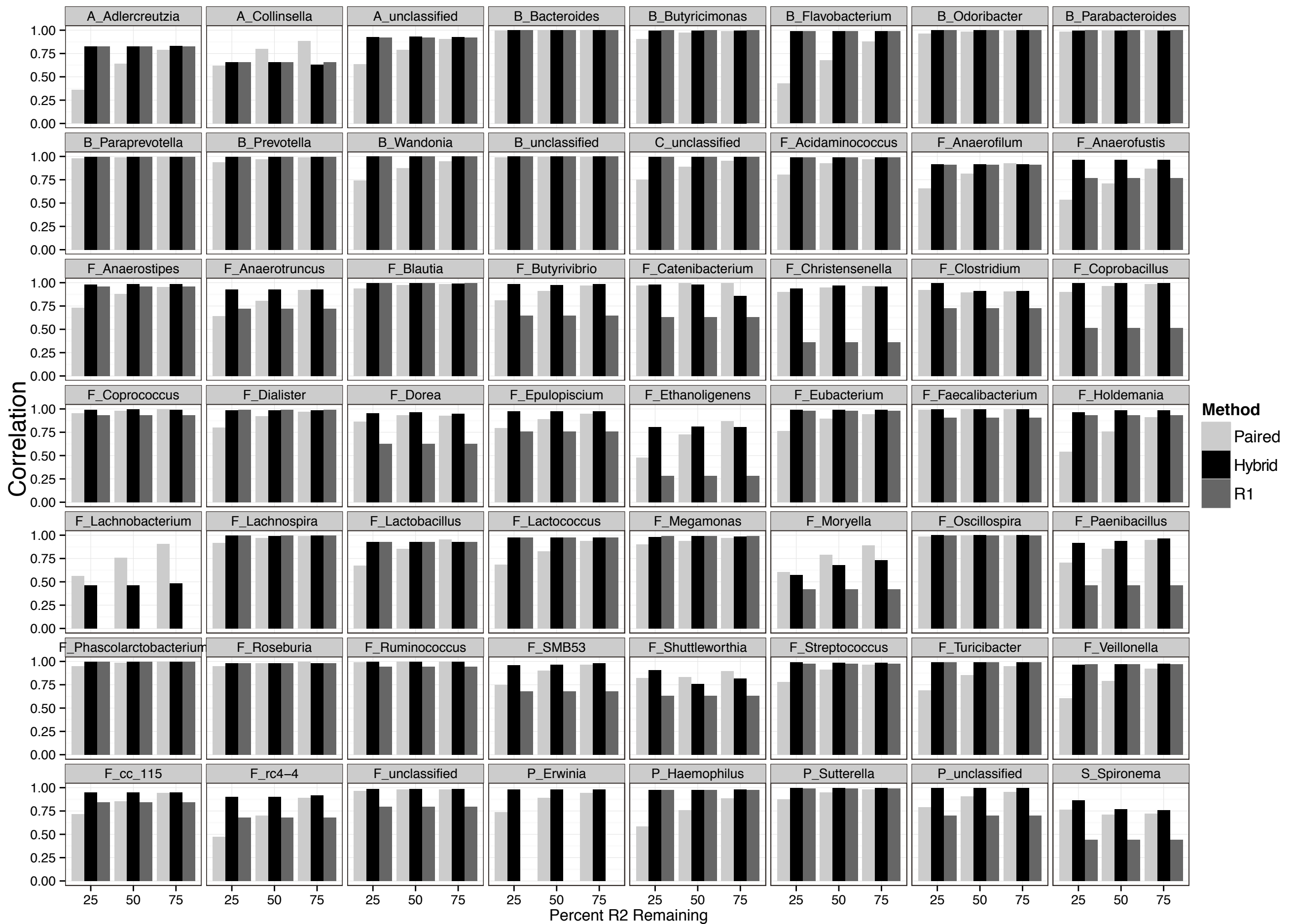

Supplement: Supplemental material [file gix129_supp.zip › SupplementaryFigure3.pdf]

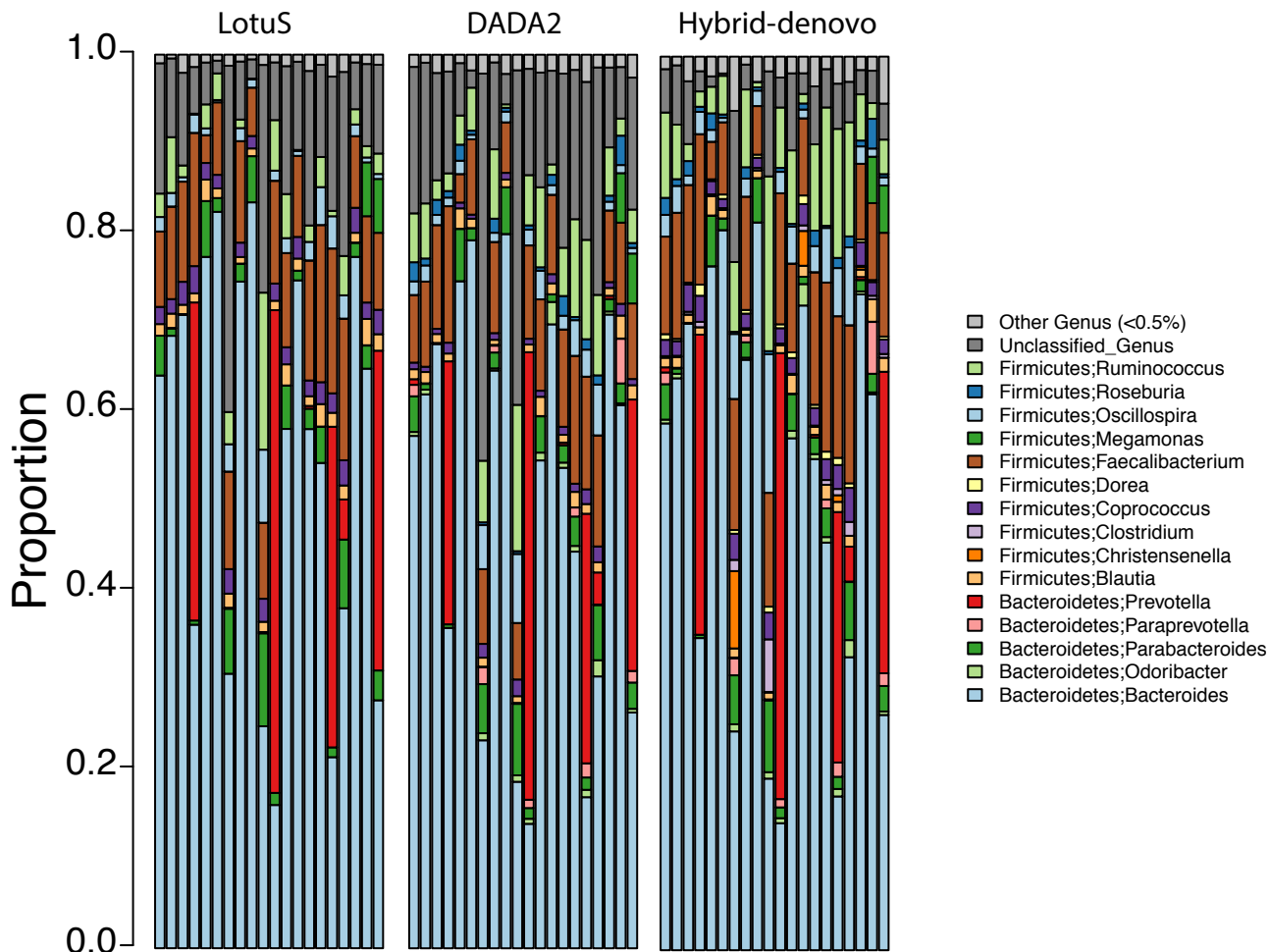

Supplement: Supplemental material [file gix129_supp.zip › SupplementaryFigure4.pdf]

**A**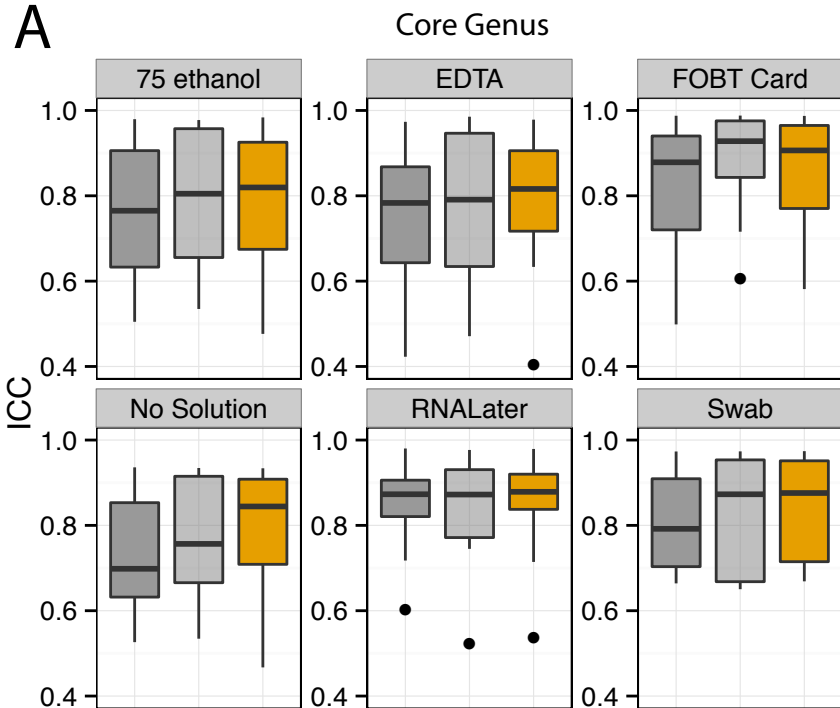**B**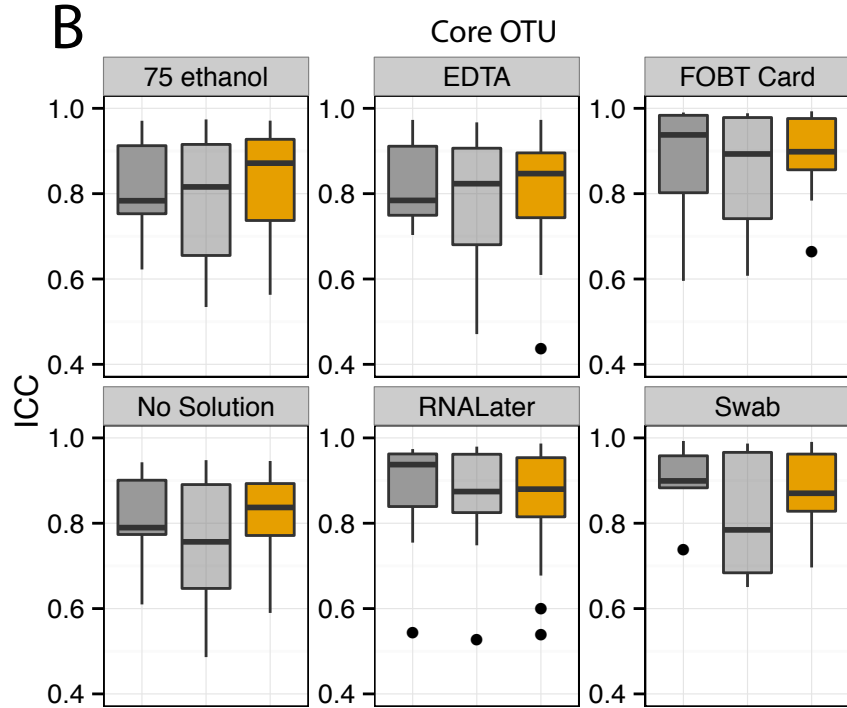

■ DADA2   ■ LotuS   ■ Hybrid-denovo

Supplement: Supplemental material [file gix129_supp.zip › SupplementaryFigure5.pdf]

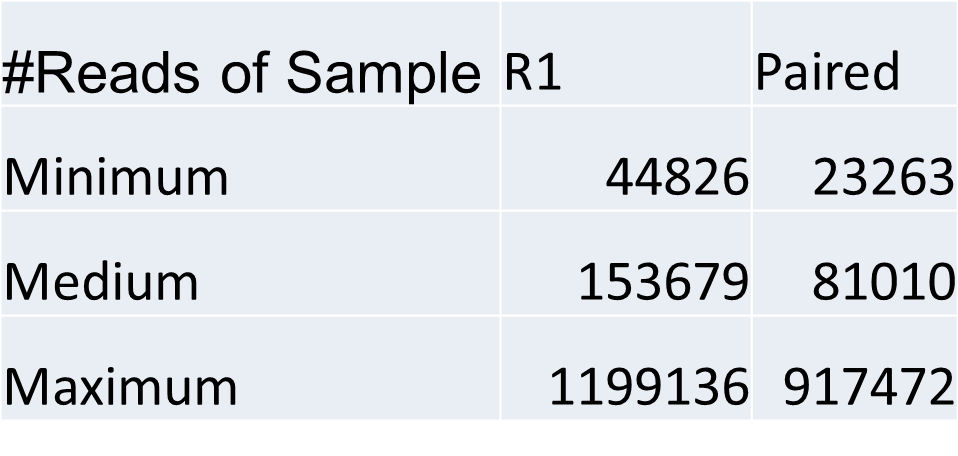

Supplement: Supplemental material [file gix129_supp.zip › SupplementaryTable1.png]
