# Supplementary material for: Hybrid-denovo: a de novo OTU-picking pipeline integrating single-end and paired-end 16S sequence tags
Source: Gigascience. 2017 Dec 15;7(3):gix129. doi: 10.1093/gigascience/gix129 (PMC5841375; doi:10.1093/gigascience/gix129)
Supplement: Response_to_Reviewer_Comments_Original_Submission.pdf [file gix129_response_to_reviewer_comments_original_submission.pdf]

Author's Response To Reviewer Comments

Dear Editor

Thanks for the acceptance of our manuscript. We have corrected some typos, modified the statement "Hybrid-denovo is more powerful than de novo OTU picking approaches based on paired-end or single-end 16S sequence tags" and uploaded LaTex source files according to your suggestion. We have also deposited the data and code on GigaDB and supplied RRID number in the manuscript.

Best,

Jun Chen & Xianfeng Chen
