# Supplementary material for: Hybrid-denovo: a de novo OTU-picking pipeline integrating single-end and paired-end 16S sequence tags
Source: Gigascience. 2017 Dec 15;7(3):gix129. doi: 10.1093/gigascience/gix129 (PMC5841375; doi:10.1093/gigascience/gix129)
Supplement: Reviewer_1_Report_(Original_Submission) -- Jeffrey Werner — 13 Nov 2017 Reviewed [file gix129_reviewer_1_report_(original_submission).pdf]

Reviewer Report

Title: Hybrid-denovo: A de novo OTU-picking pipeline integrating single-end and paired-end 16S sequence tags

Version: Original Submission **Date:** 11/13/2017

Reviewer name: Jeffrey Werner

Reviewer Comments to Author:

This work has been through several rounds of review, so I won't repeat my comments from previous rounds, which have been addressed by new comparisons with more consistent methods and more clearly documented methods. I still feel that this work presents conclusions that are valid and well-supported, although they are limited to a very specific question regarding the limitations of a low quality non-overlapping paired end HTP bacterial 16S rRNA gene sequencing survey. My only critical comment is that the authors' conclusion statement in the Abstract, "Hybrid-denovo is more powerful than de novo OTU picking approaches based on paired-end or single-end 16S sequence tags," sounds more grandiose and all-encompassing than the modest scope of the article.It seems that the authors have made additional improvements to the work in response to comments from another reviewer, and that reviewer may not be available to check those responses in this round. I've reviewed the reviewer's comments as well as the authors' responses, and changes to the manuscript. All the concerns seem to have been addressed by the authors, and I'm recommending publication.

Level of Interest

Please indicate how interesting you found the manuscript: An article whose findings are important to those with closely related research interests

Quality of Written English

Please indicate the quality of language in the manuscript: Acceptable

Declaration of Competing Interests

Please complete a declaration of competing interests, considering the following questions:

- Have you in the past five years received reimbursements, fees, funding, or salary from an organisation that may in any way gain or lose financially from the publication of this manuscript, either now or in the future?
- Do you hold any stocks or shares in an organisation that may in any way gain or lose financially from the publication of this manuscript, either now or in the future?
- Do you hold or are you currently applying for any patents relating to the content of the manuscript?

- Have you received reimbursements, fees, funding, or salary from an organization that holds or has applied for patents relating to the content of the manuscript?
- Do you have any other financial competing interests?
- Do you have any non-financial competing interests in relation to this paper?

If you can answer no to all of the above, write 'I declare that I have no competing interests' below. If your reply is yes to any, please give details below.

I declare that I have no competing interests.

I agree to the open peer review policy of the journal. I understand that my name will be included on my report to the authors and, if the manuscript is accepted for publication, my named report including any attachments I upload will be posted on the website along with the authors' responses. I agree for my report to be made available under an Open Access Creative Commons CC-BY license (http://creativecommons.org/licenses/by/4.0/). I understand that any comments which I do not wish to be included in my named report can be included as confidential comments to the editors, which will not be published.

I agree to the open peer review policy of the journal
